# Cryo-EM structure of the respiratory syncytial virus RNA polymerase

**DOI:** 10.1038/s41467-019-14246-3

**Published:** 2020-01-17

**Authors:** Dongdong Cao, Yunrong Gao, Claire Roesler, Samantha Rice, Paul D’Cunha, Lisa Zhuang, Julia Slack, Mason Domke, Anna Antonova, Sarah Romanelli, Shayon Keating, Gabriela Forero, Puneet Juneja, Bo Liang

**Affiliations:** 1 Department of Biochemistry, Emory University School of Medicine, Atlanta, GA 30322 USA; 2 Robert P. Apkarian Integrated Electron Microscopy Core, Emory University School of Medicine, Atlanta, GA 30322 USA

**Keywords:** Enzyme mechanisms, Multienzyme complexes, Virus structures, Cryoelectron microscopy, Viral infection

## Abstract

The respiratory syncytial virus (RSV) RNA polymerase, constituted of a 250 kDa large (L) protein and tetrameric phosphoprotein (P), catalyzes three distinct enzymatic activities — nucleotide polymerization, cap addition, and cap methylation. How RSV L and P coordinate these activities is poorly understood. Here, we present a 3.67 Å cryo-EM structure of the RSV polymerase (L:P) complex. The structure reveals that the RNA dependent RNA polymerase (RdRp) and capping (Cap) domains of L interact with the oligomerization domain (P_OD_) and C-terminal domain (P_CTD_) of a tetramer of P. The density of the methyltransferase (MT) domain of L and the N-terminal domain of P (P_NTD_) is missing. Further analysis and comparison with other RNA polymerases at different stages suggest the structure we obtained is likely to be at an elongation-compatible stage. Together, these data provide enriched insights into the interrelationship, the inhibitors, and the evolutionary implications of the RSV polymerase.

## Abstract (web summary)

Respiratory syncytial virus (RSV) is a pathogenic non-segmented negative-sense RNA virus and active RSV polymerase is composed of a 250 kDa large (L) protein and tetrameric phosphoprotein (P). Here, the authors present the 3.67 Å cryo-EM structure of the RSV polymerase (L:P) complex.

## Introduction

Nonsegmented negative-sense (NNS) RNA viruses are a class of pathogenic and sometimes deadly viruses that include rabies, Ebola, and respiratory syncytial virus (RSV)^1^. RSV infection is the leading cause of severe lower respiratory tract diseases in young children, older adults, and immunocompromised patients worldwide^2,3^. RSV initiates viral infection by delivering into the host cell a virus-specific RNA synthesis machine required for both genome replication and gene transcription^4,5^. This machine comprises the nucleoprotein (N) coated genomic RNA (N:RNA) and the RNA polymerase^6^. The catalytic core is a 250 kDa large (L) protein that catalyzes the RNA polymerization in both replication and transcription, the cap addition, and cap methylation of nascent viral mRNAs. A tetrameric phosphoprotein (P) is essential to modulate and constitute an active RNA polymerase with L^4,5^.

RSV RNA synthesis is believed to follow the “start-stop model” of sequential and polar transcription^7–9^. Like all NNS RNA viruses, the RSV RNA template is N:RNA, not RNA alone. The leader (Le) or trailer complementary (TrC) sequences from the terminus of the RNA genome or antigenome serve as the promoters for the RSV RNA synthesis^10–13^. To copy the N:RNA template, L requires the tetrameric P to displace N^14^. Interestingly, the RSV polymerase not only synthesizes mRNAs but also co-transcriptionally adds a cap and a poly-A tail to each transcript. The mRNA caps are synthesized using unconventional chemical reactions: (a) the cap is formed by a polyribonucleotide transferase but not a guanylyltransferase through generating a covalent L:RNA intermediate, distinct from eukaryotes and all other virus families^9,15–18^; and (b) the cap is methylated at the 2′-O position first, followed by the N-7 position, the opposite order of mammalian mRNAs^19–21^. To date, the in vitro RNA polymerization assays of RSV and several other NNS RNA viruses have been established^13,22,23^. The RSV cap addition and cap methylation assays have not been described yet and are speculated to share similar mechanisms with vesicular stomatitis virus (VSV), of which the assays were reported^16–18,24,25^.

**Fig. 1:**
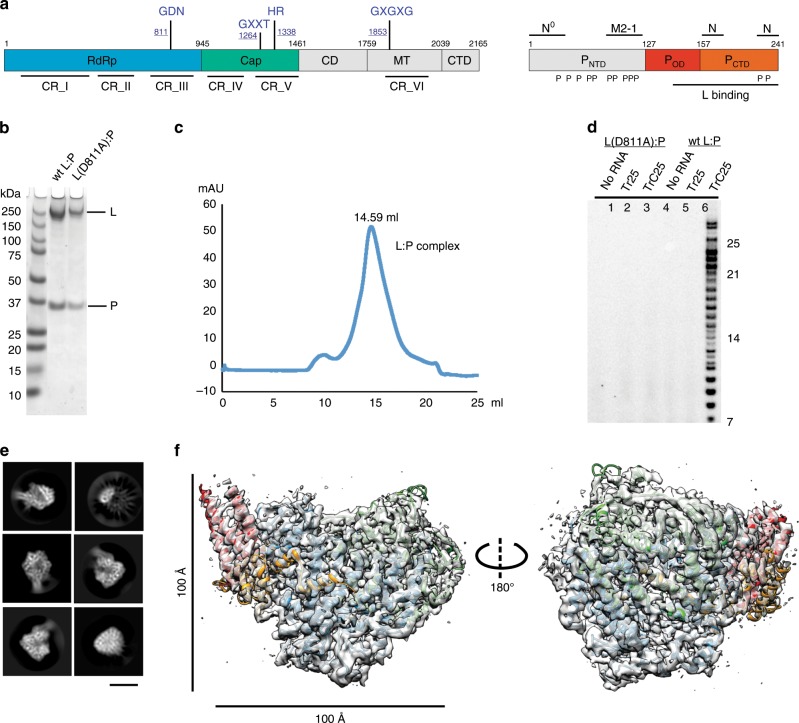
Biochemical characterization and cryo-EM structure determination. **a** Schematic domain representation of the human respiratory syncytial virus (RSV) RNA polymerase (L:P complex), with labeled domain boundary (Alignment details in Supplementary Fig. 4). The domains with missing density are colored in gray. **b** The SDS-PAGE gel shows the quality of the wild-type (wt) and mutant RSV RNA polymerase, wt L:P, and L(D811A):P, respectively (repeated ≥ 5 times). **c** Elution profile of the purified L:P complex on the Superose 6 Increase 10/300 GL size-exclusion column (repeated ≥ 5 times). **d** The RNA dependent RNA polymerization assays show that the purified wt polymerase is active and can synthesis RNA with specific RNA templates (lane 6) compared with that of catalytically inactive polymerase L(D811A):P (lane 3). **e** Representative 2D class averages from the 200 kV cryo-EM dataset, selected amongst 125 classes. Scale bar: 100 Å. **f** The final cryo-EM density map with the model (colored as **a**) in two orientations.

There are six conserved regions and three functional domains shared within L of NNS RNA viruses^26,27^. The domain boundaries and the active sites of the three functional domains, RNA dependent RNA polymerization (RdRp), capping (Cap), and cap methyltransferase (MT), are highlighted as GDN, HR, and GXGXG (X denotes any residues) with numbers, respectively^28–31^ (Fig. 1a). P is predicted to contain several disordered regions and multiple phosphorylation sites^32–38^, and P is a tetrameric protein that regulates the RNA synthesis through interacting with L, RNA-free N (N^0^), N, and M2-1 (Fig. 1a). The precise interactions between L and P and the RNA synthesis mechanism by the RSV polymerase remain poorly understood^39^. Here, we characterize the structure of the RSV polymerase (L:P) using cryo-electron microscopy (cryo-EM). Our studies reveal that the RdRp and Cap domains of the RSV L shares similar architectures of that of the VSV L^40^, uncover a previously unknown basis of how P interacts with L, and provide molecular insights into RNA synthesis by the RSV polymerase.

## Results

### Cryo-electron microscopy structure determination

We co-expressed and co-purified the recombinant RSV polymerase (L:P) from Sf21 insect cells. Gel filtration and SDS-PAGE indicated pure and full-length wild-type (wt) L:P and mutant L(D811A):P complexes (Fig. 1b, c). Using an established RdRp assay^13,41^, we demonstrated the characteristic RNA products of both de novo initiation (≤25 nt) and back primer (>25 nt) activities by the wt polymerase L:P (lane 6), but not the catalytically inactive polymerase L(D811A):P (lane 3) using a short trailer complementary 25 (TrC25) RNA template. The Le or TrC sequences of the genome serve as the promoters for the RSV polymerase. The trailer 25 (Tr25) sequence, which is the transcription product of TrC25, is not a natural template and was included as a negative control. As expected, we did not observe RNA products when using Tr25 as a template (lane 5) (Fig. 1d). Therefore, the prepared wt RSV polymerase is stable and catalytically active.

Cryo-EM analysis was conducted using a 200 kV Talos Arctica microscope with a BioQuantum/K2 direct electron detector. An initial dataset of 1349 movies resulted in 4.3 Å reconstruction, and one additional dataset of 1251 movies was collected. A total of 2600 movies gave rise to the final 3.67 Å map, refined with 253,372 particles selected from multiple rounds of 2D and 3D classifications (Fig. 1e, f, Supplementary Figs. 1–2, and Supplementary Table 1).

We performed an atomic model building on the final 3.67 Å map with COOT, assisted by the structure of the VSV L protein^40^ (PDB: 5A22). We refined the model coordinates with PHENIX. The cryo-EM map revealed the characteristic ring-like core RdRp domain and an unconventional Cap domain of the L protein that only exists in NNS RNA viruses. The map also showed the typical helix bundles of the oligomerization domain of P (Fig. 1f). Further data analysis suggested intrinsic flexibility and structural rearrangements could be attributed to the missing densities of L and P domains and will be discussed in later sections.

### The overall structure of the RSV polymerase complex

**Fig. 2:**
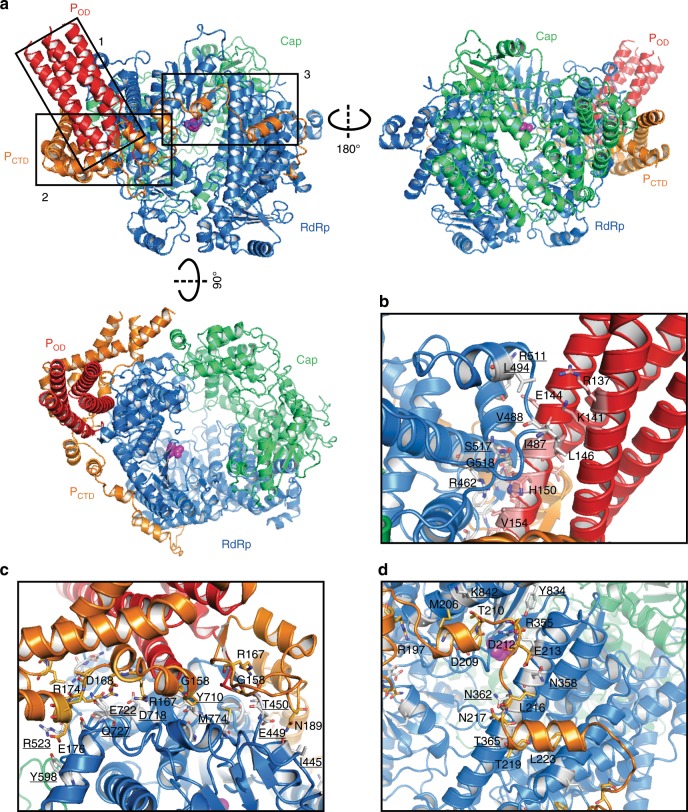
Cryo-EM structure and interactions of RSV polymerase (L:P). **a** The cartoon diagrams of RSV polymerase complex is shown in three orientations. Domains are color-coded as in Fig. 1a, and the RdRp active site (D811) is shown in the magenta sphere. The boxes indicate the magnified views of the interactions between L and P. Magnified views of interaction interfaces between the L and P_OD_ (**b**), L and P_CTD_ (**c**, **d**), and representative residues are shown. The same color scheme for the cartoons and side chains. The interacting residues of L on P are underlined.

The final atomic model contains the RdRp domain (blue) and the Cap domain (green) of the RSV L protein, and the oligomerization domain (P_OD_, red) and C-terminal domain (P_CTD_, orange) of tetrameric P proteins (Fig. 2a, representative model fitting with the map, Supplementary Fig. 3). We have assigned residues to domains as follows: RdRp, 1-945; Cap, 946-1461; P_OD_, 128-157; P_CTD_, 158-241. The RdRp domain adopts a conventional “fingers-palm-thumb” right-hand fold as many other RNA and DNA polymerases. The Cap domain that is distinct from host cells or other types of viruses shares a similar architecture as that of VSV L^40^. P_OD_ is fully assembled into a four-helix bundle as expected. Most P_CTD_ is flexible, and only the regions that interact with L can be visualized and modeled (Fig. 2a).

Sequence alignments and secondary structure predictions suggest that the RSV L shares five well-organized domains (two domains visible and modeled in this study) among NNS RNA viruses, while the P protein is flexible and contains many disordered regions^21,32–34,42–45^ (Supplementary Fig. 4). The SDS-PAGE gel (Fig. 1b) and mass spectrometry (Supplementary Fig. 5) showed that both L and P proteins are intact. However, the 3.67 Å cryo-EM map revealed no extra density for the connector domain (CD), the MT domain and the C-terminal domain (CTD) of L as well as the N-terminal domain (NTD) of P. We speculate that the missing density of L is due to significant structural rearrangements of L, and the missing density of P is primarily due to the intrinsic flexibility of P.

### Structural insights into RSV L and P interactions

The RSV L primarily uses the “fingers” motif to interact with P_OD_ and P_CTD_. The interactions between L and a tetrameric P can be divided into three parts: (1) two of four-helix bundles of P_OD_ and the RdRp; (2) two flexible P_CTD_ chains wrap around the surface of RdRp and stabilize the base of the tetrameric P_OD_; (3) one flexible P_CTD_ chain extends to the positively charged “palm” motif of RdRp and the edge of the putative NTP entrance channel (Fig. 2b–d). These interactions bury surface area of about 1101.1 Å^2^, 1542.9 Å^2^, and 884.6 Å^2^, respectively. P is negatively charged overall, and the calculated isoelectric points of P_OD_ and P_CTD_ are 4.82 and 4.34, respectively. Indeed, the interaction between L and P is dictated partly by electrostatic complementarity (Supplementary Fig. 6).

Our structure of the RSV L:P agrees with previous biochemical studies that P_CTD_ is critical to interact with L and also identifies the previously unknown role of P_OD_ in interacting with L^32,43^ (Fig. 2). On the P_OD_, residues that interact with L include K141, E144, H150, and V154 (Fig. 2b). Interestingly, four chains of P_CTD_ adopt four different conformations and interact broadly with the RdRp domain of L (Fig. 2b–d and Supplementary Fig. 6). There are extensive interactions between L and two of the P_CTD_ chains near the P_OD_, including residues G158, R167, D168, R174, E176, and N189 (Fig. 2c). There is a composite surface on the L protein that accommodates one chain of P_CTD_, and these residues serve as a strong base to stabilize the P_OD_ four-helix bundles. One P_CTD_ chain extends and uses residues D209, T210, D212, L216, T219, and L223 to interact with L (Fig. 2d). The observations of such extensive interactions are consistent with the conserved regions by the sequence alignments of L and P, respectively (Supplementary Fig. 4).

### Structural comparison of the L and P proteins

**Fig. 3:**
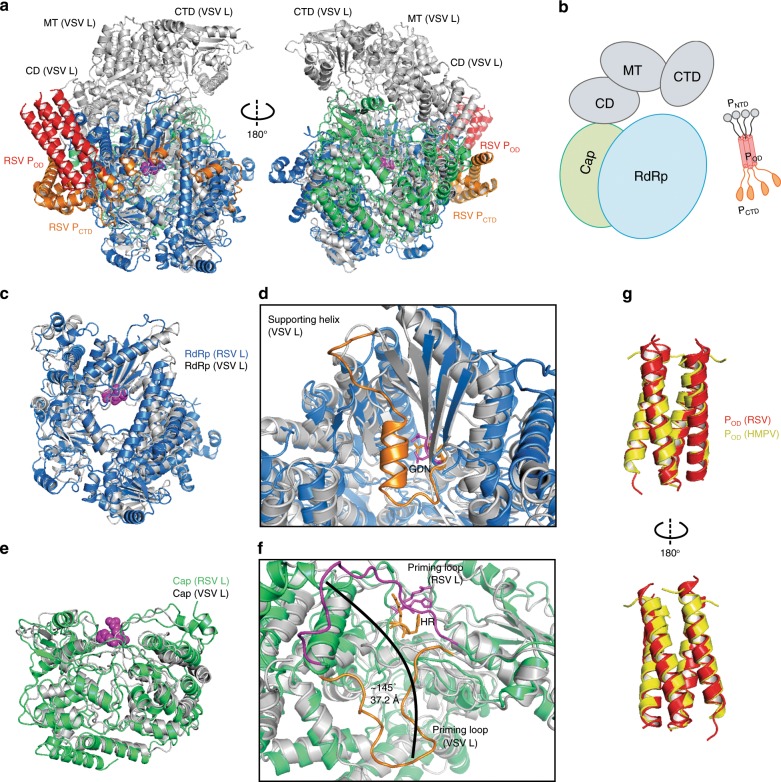
Structural comparison of the L and P proteins. **a** Superimposition of the RNA dependent RNA polymerase (RdRp) and capping (Cap) domains of the L protein from RSV (this work) and VSV (PDB: 5A22). RSV L is colored the same as Fig. 2a, and VSV L is colored in gray. **b** The cartoon representation of the RSV L and P. The missing domains are colored in gray. **c** Overview of the RdRp domain. **d** Magnified view showing the core residues (GDN, magenta for RSV, orange for VSV) and the missing supporting helix (orange, VSV L) in the RdRp domain. **e** Overview of the Cap domain. **f** Magnified view showing the core residues (HR, magenta for RSV, orange for VSV) in the Cap domain. The priming-loop-like element of the RSV L and the VSV L is highlighted in magenta and orange, respectively. A major shift (~37 Å) from the tip of the priming-loop-like element is shown as the black arrow. **g** Superimposition of the oligomerization domain of P (P_OD_) from RSV (this work) and HMPV (PDB: 4BXT). The HMPV P is in gold.

When the structure of the RSV L:P overlays with that of the VSV L^40^ (PDB: 5A22), the CD, MT, and CTD domains (gray) extends from the top part of the complex (Fig. 3a, b). The RSV RdRp domain is comparable to many other RNA polymerases^40,46–51^ (Fig. 3c and Supplementary Fig. 7). The RSV Cap domain exhibits a similar overall fold to that of VSV L^40^ (Fig. 3e and Supplementary Fig. 8).

However, there are notable differences: (1) There are additional motifs at the N-terminal regions of the RSV RdRp; (2) The RSV PdRp has a missing connecting helix (residues 660–691) (equivalent to VSV residues 571–597) adjacent to the active site (Fig. 3d and Supplementary Fig. 8); (3) The RSV priming-loop-like element (residues 1265–1282, magenta) of the Cap domain shows a significant shift of 146° and 37.2 Å compared to its equivalent motif of VSV L (residues 1155–1174, orange), and the pivot points for the shift are the residues 1264 and 1283 (refs. ^40,50,52^) (Fig. 3f and Supplementary Fig. 8).

We also compared the structures of the RSV P with P from other NNS RNA viruses. P is the most variable protein and shares the lowest sequence identities among NNS RNA viruses. Strikingly, despite the low sequence conservation, all P (or VP35 in *Filoviridae*) share a common feature and exist as an oligomer (dimer, trimer, or tetramer) in solution^53–60^ (Fig. 3g and Supplementary Fig. 9). Although the precise role of the oligomerization of P remains unclear, the comparison suggests the oligomerization of P plays critical roles in enhancing the interactions with L and bridging multiple sets of co-factors (i.e., N and M2-1) during RNA synthesis.

### Conformational transitions of L:P

The significant flexibility of P leads to a weaker density for many regions of P (visualized at a lower σ). Further 3D classification revealed an almost equal number (63,912 vs. 82,400) of the particles that show less P density and yielded a cryo-EM map at 4.86 Å resolution. Compared with a 4.54 Å map, the RdRp and Cap domains of L are virtually identical, but the density for P is almost depleted (Supplementary Fig. 10). Focused refinement of P regions does not yield a higher resolution map, confirming the intrinsic flexibility and mobility of P.

The substantial conformational variability of CD, MT, and CTD of L leads to a missing density throughout the initial image processing. A larger size 3D class produced a 7.2 Å resolution map of reconstruction. Additional density blobs appear on the top part of the polymerase, agree with the potential location of the missing domains. However, no well-defined density for these domains was observed in the cryo-EM map (Supplementary Fig. 10).

## Discussion

This work provides structural insights into the polymerase (L:P) of RSV, a significant NNS RNA virus pathogen, and offers a framework for understanding the coordination of the enzymatic activities of L within structurally distinct but functionally coordinated domains. Further investigations are required to discover whether the RSV L alone exhibits the same way as VSV L. Interestingly, all RNA polymerases of NNS RNA viruses require P (or VP35 in *Filoviridae*), but P exists as diverse oligomeric protein (Supplementary Fig. 9) and shares low sequence identity^53–60^. In most cases, the protomers, the basic building blocks of oligomeric proteins, assemble into a higher-order oligomer following a defined repeating or symmetry rule. However, the nonsymmetric structures of P_CTD_ suggest that P likely plays a nonsymmetric structural role more than previously appreciated. Except for the P_OD_, every chain of the P tetramer appears to either interact with different sites of L, such as the “palm” motif of the RdRp domain, which may be important for regulating the polymerase activities or remain flexible waiting for interacting with other binding partners. These pronounced structural differences attest to a high degree of versatility in L upon binding of P.

**Fig. 4:**
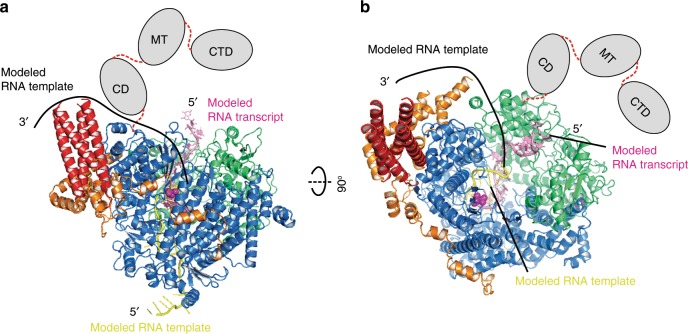
The proposed model of RSV RNA synthesis. **a**, **b** The RSV polymerase with modeled RNA template and transcript (PDB: 6QCV). The same color scheme for RSV proteins as Fig. 2a. The modeled RNA template and RNA transcript are shown in yellow and pink, respectively. (Supplementary Fig. 11 for additional comparisons). The 3′ RNA template enters the RdRp from the bottom, and 5′ RNA transcript exits from the top. The upstream 3′ RNA template is drawn in black line.

Indeed, it was suggested that L bears much movement during RNA synthesis. We compared the priming-loop-like element of the RSV L to that of the VSV L (Supplementary Fig. 8). The priming-loop-like element of the VSV Cap domain has been demonstrated to play critical roles in transcription initiation and capping^61^, and it was thought to be at the initiation stage because the overlay of the structure of the initiation complex of other RdRP (such as reovirus polymerase) revealed that this priming-loop-like element of the VSV L occupied the same location as the priming loop in the reovirus polymerase^40,50^. In addition, the comparisons of the VSV L and the initiation/elongation complex of the reovirus polymerase suggest that if the RNA product extends, there will be not enough space to accommodate additional newly synthesized RNA products, and this priming-loop-like element is likely to move away from this initiation stage^40,50^ (Supplementary Fig. 11c–d). We also superposed the elongation complexes of other RdRPs (such as FluB, polio, or rotavirus polymerase) with the RSV L:P, and the position of the priming-loop-like element does not “conflict” with the locations of either RNA template or RNA product^52,62,63^ (Supplementary Fig. 11). Together, the missing connecting helix (residues 660–691) of the RSV RdRp domain and the significant shift of the priming-loop-like element of the RSV Cap domain suggest that the structure we obtained is likely to be at an elongation-compatible stage. Based on the structures of the VSV L (preinitiation stage, PDB: 5A22) and influenza polymerase (elongation stage, PDB: 6QCV), we modeled the RNA template and the transcript into our RSV polymerase structure^40,50,52,62,63^ (Fig. 4a, b and Supplementary Fig. 11).

Besides, our study has implications for understanding current RSV polymerase inhibitors. Previous studies showed that resistance to the nucleoside analog inhibitor ALS-8112 is conferred by QUAD resistance mutations (M628L, A789V, L795I, and I796V) in the RdRp domain of L, while a nonnucleoside inhibitor AZ-27 that inhibited the RdRp activity could be escaped by a Y1631H resistance mutation^64^. In our structure, the QUAD mutation sites (yellow) of ALS-8112 are in approximate close location to the active site (D811, magenta), and the QUAD mutations can potentially alter the microenvironment of RNA synthesis and the conformation of the RdRp active sites (Supplementary Fig. 12). However, Y1631 is not visible in the structure, and the inhibition mechanism of AZ-27 remains unclear.

Further, our study has evolutionary implications: How did three distinct enzymatic activities (RNA polymerization, RNA capping, and RNA cap methylation) for RNA synthesis integrate within a single polypeptide (L)? How are those functional domains evolved/related to multiple much complex counterparts in the eukaryotic cell and many other viruses? Further studies will reveal whether this “compact” mechanism illustrates the evolutionary pressure applied for the RNA synthesis machinery in general.

During the review process of our manuscript, another structure of the RSV polymerase (L:P) complex in an *apo* state was published^65^. We superposed the published structure (PDB: 6PZK) with our structure (PDB: 6UEN) described here, and they share similar overall fold with high similarities (RMSD = 1.450 Å). Interestingly, the superpositions of the individual RdRp domain, Cap domain, and the P tetramers yield lower RMSD values of 1.240, 1.021, and 0.991 Å, respectively. The structural comparisons suggest that the individual domains are mostly the same, but the inter-domain arrangements of the two structures have slight differences. In the closer dissection of both structures, we identified minor shifts between the interface of the L:P complex, in particular, the P tetramers and the two helixes of L that interact with P (dock A and dock B) if fixed the position of the RdRp domain. It appears that the P tetramers slide closer to L, and the docks A and B shift towards the RdRp domain and adopt more compact packing to accommodate closer interactions with P (6PZK) than that of this work (6UEN) (Supplementary Fig. 13 and arrows indicate the shift direction). This observation suggests plasticity in the L:P interaction, and that this interface may adopt a larger degree of conformational rearrangements during RNA synthesis.

Outstanding questions remain: (1) It is known that the phosphorylation of P regulates the activities of L. Previous studies suggested many potential phosphorylation sites reside in the P_NTD_ (1–127) but are not visible in this study^36–38^. (2) How other co-factors such as M2-1, N^0^, or N:RNA influence (or stabilize) the polymerase conformations? To determine the structures of L:P:RNA or L:P:M2-1 complexes may be a future research focus.

## Methods

### Expression and purification of the RSV polymerase (L:P)

Codon-optimized RSV (strain A2) L and P genes (the DNA sequences and primers are listed in Supplementary Table 2) were subcloned into the pFastBac Dual vector (MacroLab), and the virus was prepared using the Bac-to-Bac system (Invitrogen). PCR-based site-directed mutagenesis was used for the construction of mutant L(D811A) with the plasmid encoding the wt L:P complex as the template (the primers are listed in Supplementary Table 2c). N-terminal 6x His-tagged L containing a TEV cleavage site and no-tagged P was co-expressed in baculovirus-mediated transduction of Sf21 suspension cell cultures. Cells were lysed by Dounce homogenization in lysis buffer (50 mM sodium phosphate pH 7.4, 300 mM NaCl, 6 mM MgSO_4_, 10% glycerol, 0.2% NP-40, and EDTA-free protease inhibitor), followed by Co^2+^-NTA agarose beads (GoldBio), washed with wash buffer (50 mM sodium phosphate pH 7.4, 300 mM NaCl, 6 mM MgSO_4_, 10% glycerol, and 10 mM imidazole), and eluted with elution buffer (50 mM sodium phosphate pH 7.4, 300 mM NaCl, 6 mM MgSO_4_, 10% glycerol, and 250 mM imidazole). The eluted sample was treated with TEV enzyme in TEV cleavage buffer (50 mM sodium phosphate pH 7.4, 300 mM NaCl, 6 mM MgSO4, 10% glycerol, and 0.2% NP-40, 1.4 mM 2-Mercaptoethanol) overnight at 16 °C and further applied to Co-NTA. The flow-through sample was applied to the Heparin column (Buffer A: 50 mM Tris-HCl pH 8.0, 50 mM NaCl, 10% Glycerol, and Buffer B: 50 mM Tris-HCl pH 8.0, 1.5 M NaCl, 10% Glycerol) and followed by size-exclusion chromatography using the gel filtration buffer (25 mM HEPES pH 7.4, 300 mM NaCl, 6 mM MgSO_4_, and 0.5 mM TCEP) with a Superose 6 Increase 10/300 GL (GE Healthcare). The quality of purified proteins was analyzed by SDS-PAGE gel. The bands migrating ~250 and ~35 kDa were confirmed to be RSV L and P polypeptides by liquid chromatography-mass spectrometry (LC/MS, Supplementary Fig. 5). The proteins were tested active for RNA synthesis activity. The pure proteins were flash-frozen in liquid nitrogen and stored in 30 μl aliquots at −80 °C for further use. The mutant L(D811A):P complex was expressed, purified, and stored in the same manner as the wt L:P complex.

### In vitro RNA synthesis assay

The terminal trailer complementary (TrC25: 5′-UAGUUUUUGACACUUUUUUUCUCGU-3′) template and trailer (Tr25: 5′-ACGAGAAAAAAAGUGUCAAAAACUA-3′) product sequences of the RSV genome were used in the in vitro RdRp assay (detailed procedures described in Noton et al.^13^). RNA oligonucleotides were chemically synthesized from IDT. Radioactive isotope-labeled nucleotides [α-^32^P] GTP was purchased from Perkin Elmer. The reaction mixtures contained 2 µM RNA template, RSV L:P complexes (~300 ng RSV L), NTPs (ATP, CTP, and UTP each at 1.25 mM and GTP at 50 µM with 5 µCi of [α-^32^P]GTP), and reaction buffer (50 mM Tris-HCl at pH 7.4, 8 mM MgCl_2_, 5 mM DTT, 10% glycerol) in a final volume of 20 µl. The reactions were incubated at 30 °C for 5 h, heated to 90 °C for 5 min, and then stopped by adding 5 µl stop buffer (90% formamide, 20 mM EDTA, 0.02% bromophenol blue). The RNA products were analyzed using a 20% polyacrylamide gel containing 7 M urea in a TBE buffer, followed by autoradiography. The RNA ladder used was generated by incubating [ɣ-^32^P] ATP and 7-nt, 14-nt, 21-nt, and 25-nt RNA trailer sequences (Tr7, Tr14, Tr21, and Tr25) with T4 PNK (NEB). The sequences of the RNA oligos used to generate the ladders are as follows: Tr7 (5′-ACGAGAA-3′), Tr14 (5′-ACGAGAAAAAAAGU-3′), Tr21 (5′-ACGAGAAAAAAAGUGUCAAAA-3′), and Tr25 (5′-ACGAGAAAAAAAGUGUCAAAAACUA-3′).

### Liquid chromatography-tandem mass spectrometry (LC-MS/MS)

Protein samples (~3 μg) were treated with 1 mM dithiothreitol (DTT) at room temperature (RT) for 30 min, followed by 5 mM iodoacetamide at RT for 30 min in the dark. Proteins were digested overnight with 0.5 μg lysyl endopeptidase (Wako) at RT and further digested overnight with 1 μg trypsin (Promega) at RT. Resulting peptides were desalted with an HLB column (Waters). Peptides were analyzed with a Q Exactive™ Plus Hybrid Quadrupole-Orbitrap™ Mass Spectrometer (Thermo Fisher Scientific). Mass spectrometry data were analyzed using Proteome Discoverer 2.1 against the UniProt database of human respiratory syncytial virus A (strain A2) and *Spodoptera frugiperda* (Fall armyworm).

### Cryo-EM sample preparation and data acquisition

A total of 3.0 μl of the purified RSV polymerase (L:P) complex at a concentration of 0.33 mg/ml were applied to a glow-discharged Quantifoil holey carbon grid (R1.2/1.3, Cu, 400 mesh) (SPI). Grids were blotted for 3 s at ~90% humidity at RT and plunge-frozen in liquid ethane using a Cryoplunge 3 System (Gatan). Cryo-EM data were recorded on a Talos Arctica 200 kV (TEM) with BioQuantum/K2 direct electron detector (Thermo Fisher) at Emory University. All cryo-EM movies were recorded in counting mode using EPU (Thermo Fisher). The nominal magnification of ×130,000 corresponds to a calibrated pixel size of 1.045 Å on the specimen. The dose rate was set to 1.365 e^-^/Å^2^/frame. The total exposure time of each movie was 10 s, leading to a total accumulated dose of 55 e^−^/Å^2^, fractionated into 40 frames (250 ms per frame). All movies were recorded in a defocus range between −1.25 and −2.5 μm. A total of 2600 micrographs were collected from two separate data collection sessions.

### Image processing and 3D reconstruction

Drift correction, beam-induced motion, and dose-weighting were performed for dose-fractionated movies by the program MotionCor2^66^ with a 5 × 5 patch, resulting in corrected movies and summed images. All 40 frames in each movie were summed with the dose-weighted scheme. The summed images were used in all image processing steps. The contrast transfer function (CTF) was estimated using the program CTFFIND4^67^. To generate RSV polymerase complex templates for automatic picking and the initial model, around 50,000 particles were manually picked and classified. The data were initially processed in two datasets, one for each data collection session (1349 and 1251 micrographs) and then merged into a total of 2600 micrographs. A total of 792,070 particles were picked, and the box size of 200 pixels was used to extract the particles. Particle picking and screening, 2D classification, as well as the initial 3D model building, 3D classification, 3D refinement, CTF refinement, and polishing were performed using RELION 3.0.7^68^. The final refinement was validated using cisTEM^69^, using the best class as the initial model. The global search was performed once without the mask followed by another global search using a soft mask (six-pixel soft edge) that was generated in RELION. All reported resolutions were based on gold-standard refinement procedures and the Fourier shell correlation (FSC) = 0.143 criterion. Local resolution was estimated using ResMap^70^.

### Model building and refinement

The 3.67 Å resolution map was used for model building and refinement. To obtain better side-chain densities for model building, B-factor was used for map sharpening. The coordinates of the VSV L protein (PDB: 5A22) and the homology models of the RSV L protein by Phyre2^71^ and I-TASSER^72^ were positioned into the map as the initial guides using UCSF Chimera^73^ and COOT^74^. The structure model was manually built by COOT. Structure factors were calculated by PHENIX^75,76^, and the full structure was subjected to multiple cycles of global real-space refinement with rotamer, Ramachandran plot restraints enabled in PHENIX. FSCs were calculated between the two half maps, the model against the working map and the other (free) half map and full (sum) map. The confidence maps and locally sharpened maps were calculated to facilitate the model building^77,78^. MolProbity^79^ was used to validate the geometries of the model.

### Figure preparation

All the figures representing model and electron density maps were generated using COOT^74^, UCSF Chimera^73^, and PyMOL^80^. Multiple sequence alignments were performed using Multalin^81^ and ESPript^82^.

### Reporting summary

Further information on research design is available in the Nature Research Reporting Summary linked to this article.

## Supplementary information

Supplementary Information

Reporting Summary

## Data Availability

The data that support the findings of this study are available from the corresponding author upon reasonable request. The 3D cryo-EM density maps and atomic coordinates generated and analyzed during the current study are available on the Electron Microscopy Data Bank (Accession code: EMD-20754) and Protein Data Bank (Accession code: 6UEN), respectively.
