# Supplementary material for: Cryo-EM structure of the respiratory syncytial virus RNA polymerase
Source: Nat Commun. 2020 Jan 17;11:368. doi: 10.1038/s41467-019-14246-3 (PMC6969064; doi:10.1038/s41467-019-14246-3)
Supplement: Supplementary file 1 — Supplementary Information [file 41467_2019_14246_MOESM1_ESM.pdf]

Supplementary information

Cryo-EM structure of the respiratory syncytial virus RNA polymerase

D. Cao *et al.*

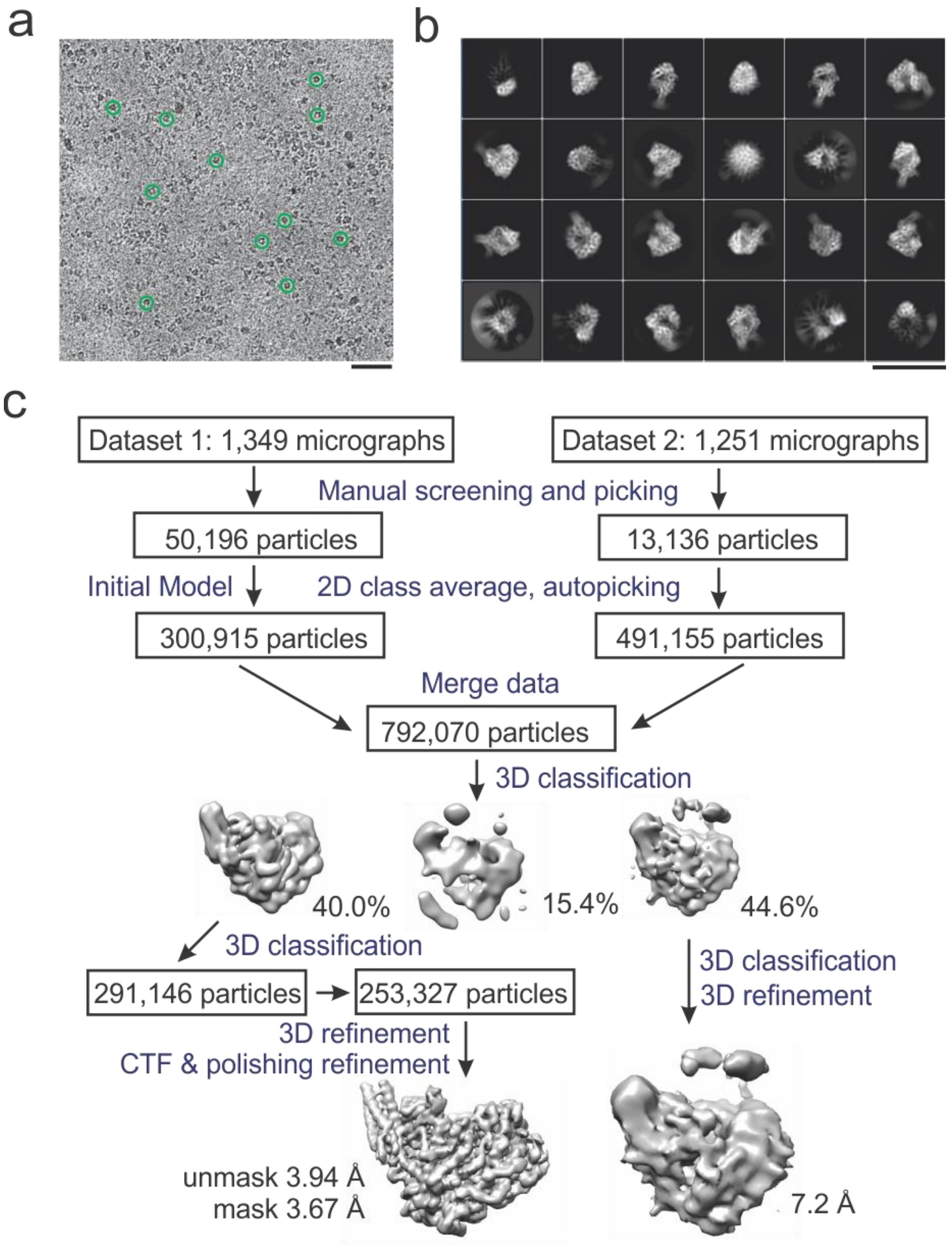

Supplementary Figure 1: Data collection and image processing. a, Representative micrograph of the respiratory syncytial virus (RSV) polymerase (L:P complex). Images were collected on a 200kV Arctica (Thermo Fisher) equipped with a BioQuantum/K2 direct electron detector. The magnification is 130,000x with a calibrated pixel size of 1.045 Å. Scale bar: 500 Å. **b**, Representative 2D class averages obtained from the reference-free 2D classification. Scale bar: 200 Å. **c**, Datasets, classification, and refinement procedures used in this study.

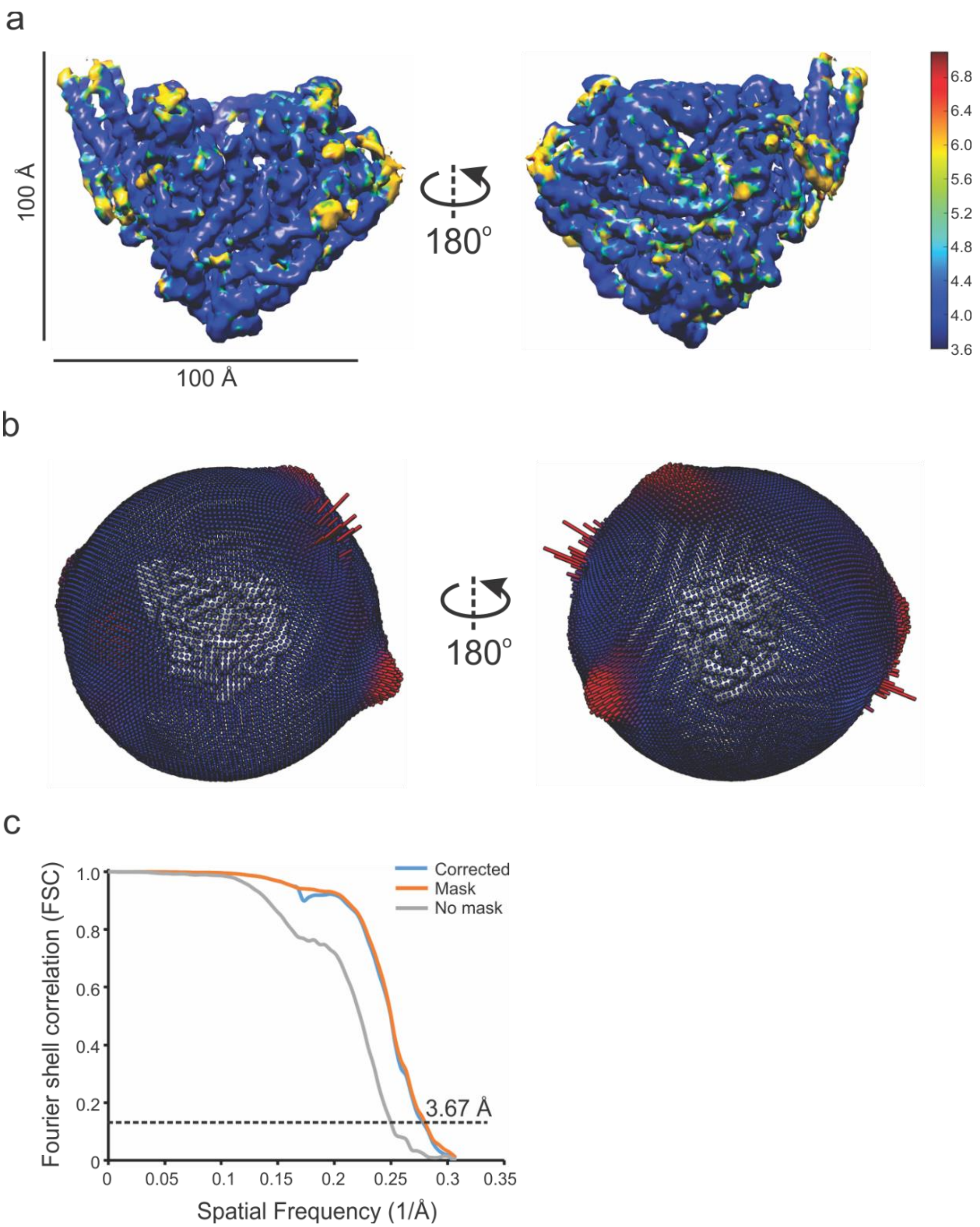

Supplementary Figure 2: Single-particle cryo-EM analysis of RSV polymerase (L:P) complex. **a**, The density map from refinement colored by local resolution estimate (calculated using ResMap) and shown. **b**, Angular-distribution histogram of particles used in calculating the final 3D reconstruction for the RSV polymerase. **c**, Gold-standard Fourier shell correlation (FSC) curves for cross-validation between the corrected, mask, and unmasked maps.

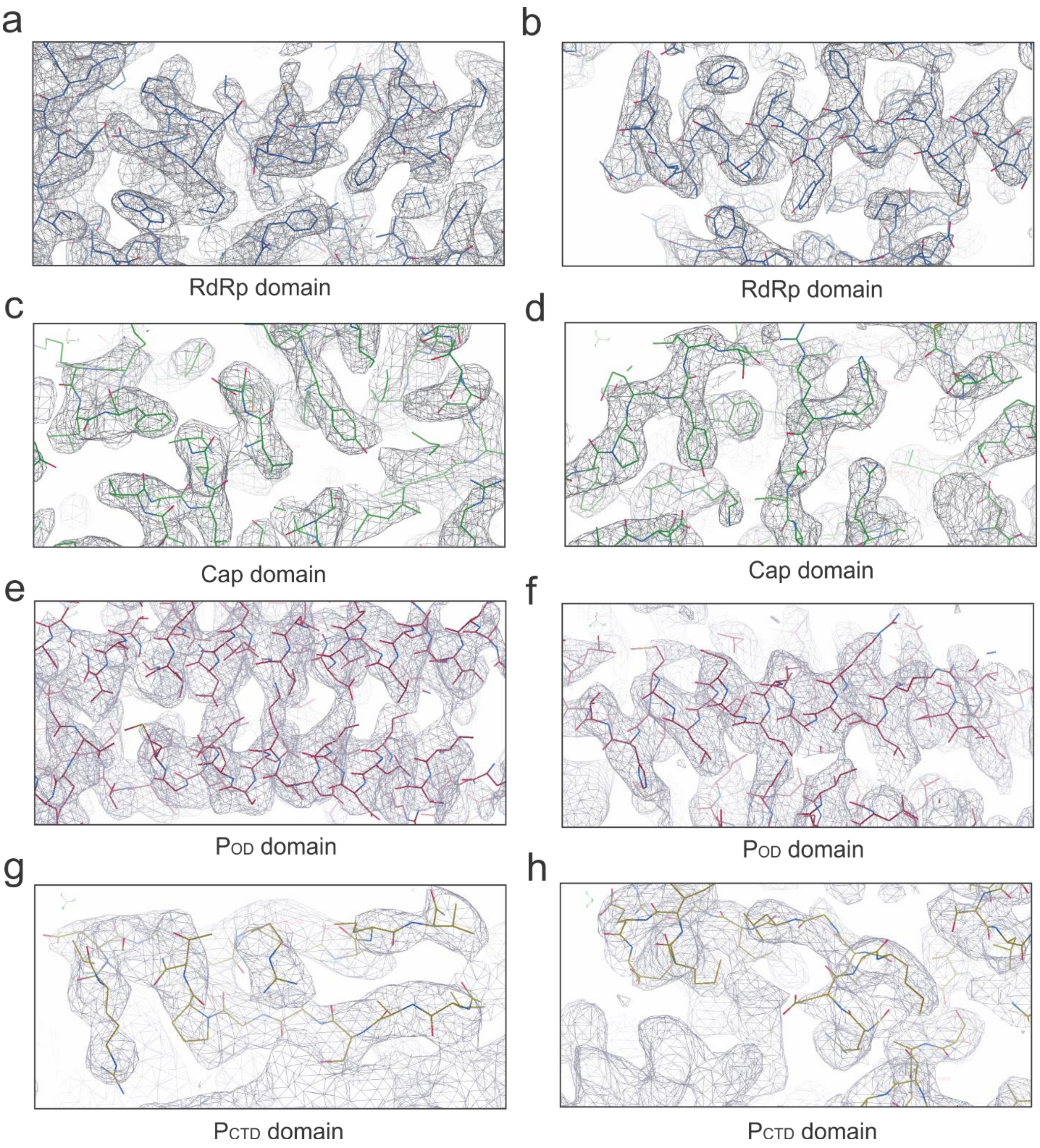

Supplementary Figure 3: The RSV polymerase (L:P) complex model and electron microscopy density. a-h, Electron microscopy density segments for representative regions of each domain, RNA dependent RNA polymerization domain (RdRp, blue, **a-b**) and the capping (Cap, green, **c-d**) domain of the RSV L protein, and the oligomerization domain (P_{OD}, red, **e-f**) and C-terminal domain (P_{CTD}, orange, **g-h**) of the RSV P protein. Sharpened map contour as details in Methods.

a Sequence alignment of the L protein

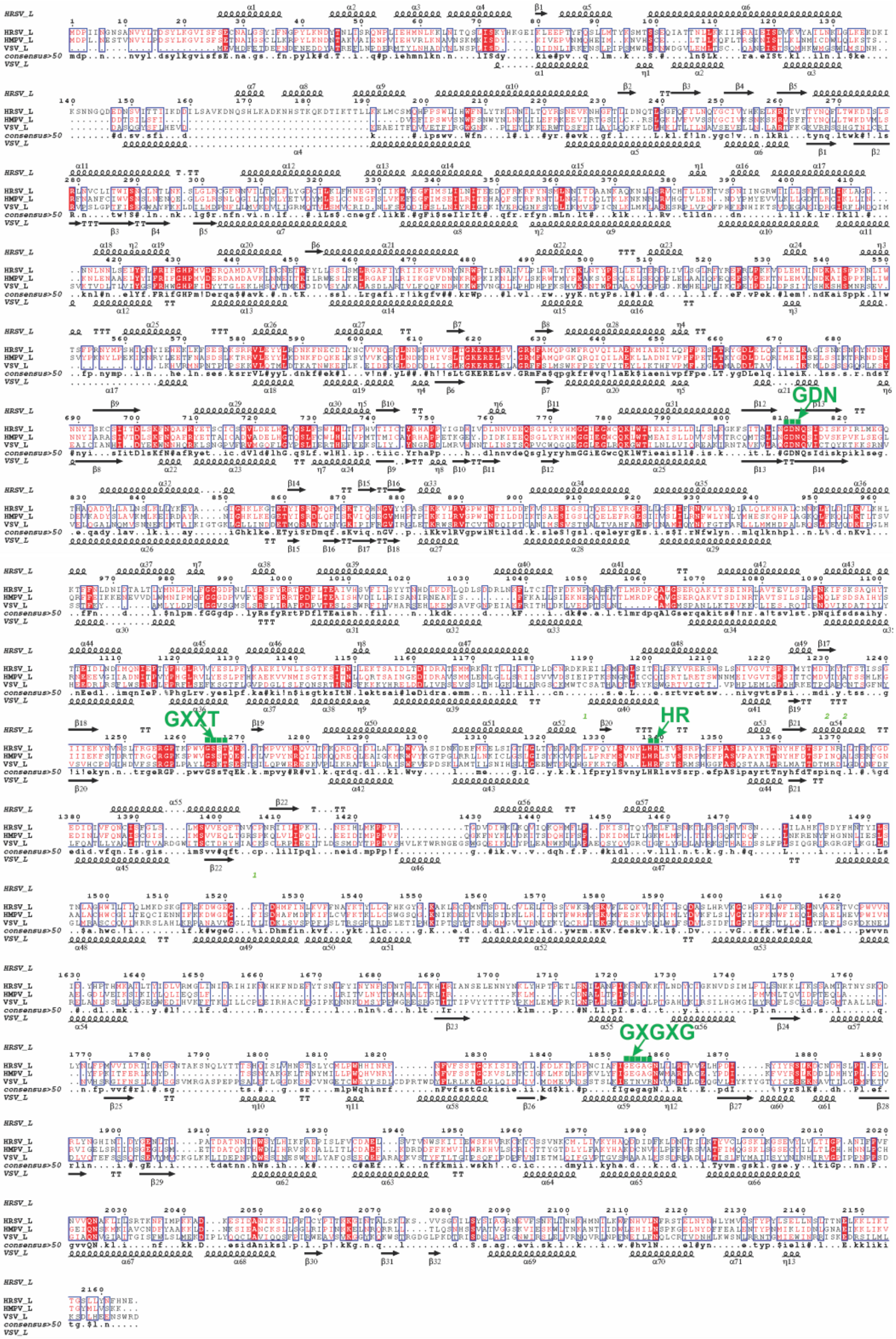

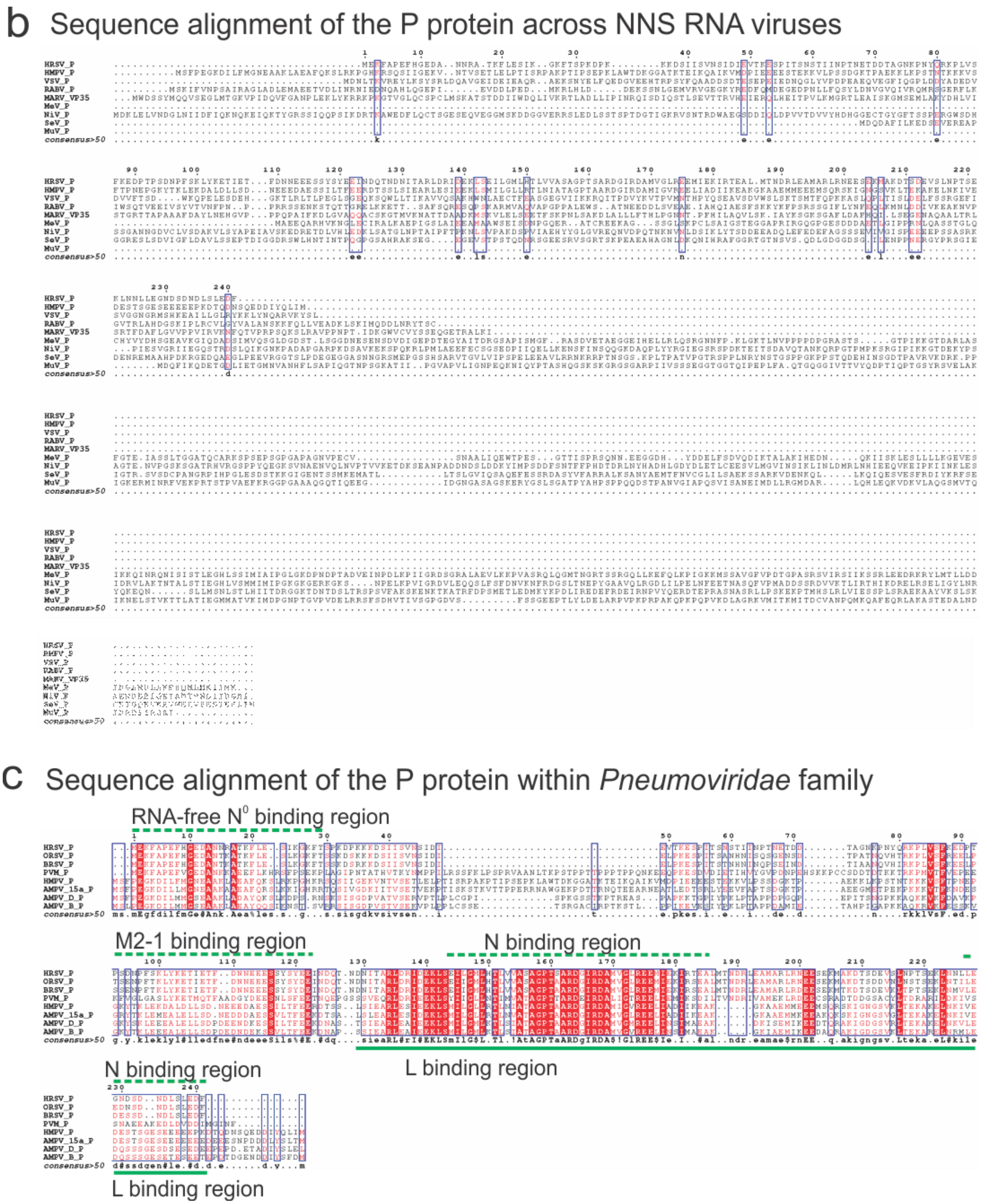

Supplementary Figure 4: Sequence alignment of the RSV L and P. The alignment graphics were prepared with Multalin and ESPript. **a**, The alignment of the L protein from Human respiratory syncytial virus strain A2 (P28887 [https://www.uniprot.org/uniprot/P28887]), Vesicular stomatitis Indiana virus strain San Juan (P03523 [https://www.uniprot.org/uniprot/P03523]), and Human metapneumovirus (Q91L20 [https://www.uniprot.org/uniprot/Q91L20]). The secondary structures of the HRSV L identified

are shown above the alignment, and the secondary structures of the VSV L (PDB: 5A22 [http://dx.doi.org/10.2210/pdb5A22/pdb]) are shown below the alignment. Catalytic residues of the RNA dependent RNA polymerase (RdRp), cap addition (capping), and cap methylation (MT) are highlighted using green squares with text. **b**, The alignment of the P protein across non-segmented negative-sense (NNS) RNA viruses. The alignment is in poor agreement, suggesting that the large variability among NNS RNA viruses. The sequences of HRSV P (X5EYT2 [https://www.uniprot.org/uniprot/X5EYT2]), Human metapneumovirus P (Q91KZ5 [https://www.uniprot.org/uniprot/Q91KZ5]), Vesicular stomatitis Indiana virus strain Mudd-Summers P (P04880 [https://www.uniprot.org/uniprot/P04880]), Rabies virus strain China/MRV P (Q0GBY3 [https://www.uniprot.org/uniprot/Q0GBY3]), Lake Victoria marburgvirus strain Musoke-80 VP35 (P35259 [https://www.uniprot.org/uniprot/P35259]), Measles morbillivirus P (Q83623 [https://www.uniprot.org/uniprot/Q83623]), Nipah virus P (D2DEB8 [https://www.uniprot.org/uniprot/D2DEB8]), Sendai virus strain Harris P (P04859 [https://www.uniprot.org/uniprot/P04859]), and Mumps rubulavirus P (Q8QY72 [https://www.uniprot.org/uniprot/Q8QY72]) are shown in the alignment. **c**, The alignment of the P protein from *Pneumoviridae* family. The alignment is in good agreement, indicating high-level similarities shared within this family. The P protein sequences from HRSV (X5EYT2 [https://www.uniprot.org/uniprot/X5EYT2]), Ovine respiratory syncytial virus strain WSU 83-1578 (Q83956 [https://www.uniprot.org/uniprot/Q83956]), Bovine respiratory syncytial virus strain A51908 (P33454 [https://www.uniprot.org/uniprot/P33454]), Pneumonia virus of mice J3666 (Q5MKN9 [https://www.uniprot.org/uniprot/Q5MKN9]), Human metapneumovirus P (Q91KZ5 [https://www.uniprot.org/uniprot/Q91KZ5]), Avian metapneumovirus strain isolate Canada goose/Minnesota/15a/2001 (Q2Y2M5 [https://www.uniprot.org/uniprot/Q2Y2M5]), Avian metapneumovirus type D (A0A077SG92 [https://www.uniprot.org/uniprot/A0A077SG92]), and Avian metapneumovirus type B (A8Y7P8 [https://www.uniprot.org/uniprot/A8Y7P8]) are shown in the alignment.

a Mass spec coverage of the RSV L protein

```
     1          11         21         31         41         51         61         71         81         91         101        111        121
   1 MDPIINGNSA NVYLTDSYLK GVISFSECNA LGSYIFNGPY LKNDYTNLIS RQNPLIEHMN LKKLNITQSL ISKYHKGEIK LEEPTYFQSL LMTYKSMTSS EQIATTNLLK KIIRRAIEIS DVKVYAILNK
 131 LGLKEKDKIK SNNGQDEDNS VITTIIKDDI LSAVKDNQSH LKADKNHSTK QKDTIKTTLL KKLMCSMQHP PSWLIHWFNL YTKLNNILTQ YRSNEVKNHG FTLIDNQTLS GFQFILNQYG CIVYHKELKR
 261 ITVTTYNQFL TWKDISLSRL NVCLITWISN CLNTLNKSLG LRCGFNNVIL TQLFLYGDCI LKLFHNEGFY IIKEVEGFIM SLILNITEED QFRKRFYNSM LNNITDAANK AQKNLLSRVC HTLLDKTVSD
 391 NIINGRWIIL LSKFLKLIKL AGDNNLNNLS ELYFLFRIFG HPMVDERQAM DAVKINCNET KFYLLSSLSM LRGAFIYRII KGFVNNYNRW PTLRNAIVLP LRWLTYYKLN TYPSLLELTE RDLIVLSGLR
 521 FYREFRLPKK VDLEMIINDK AISPPKNLIW TSFPRNYMPS HIQNYIEHEK LKFSESDKSR RVLEYYLRDN KFNECDLYNC VVNQSYLNNP NHVVSLTGKE RELSVGRMFA MQPGMFRQVQ ILAEKMIAEN
 651 ILQFFPESLT RYGDLELQKI LELKAGISNK SNRYNDNYNN YISKCSIITD LSKFNQAFRY ETSCICSDVL DELHGVQSLF SWLHLTIPHV TIICTYRHAP PYIGDHIVDL NNVDEQSGLY RYHMGGIEGW
 781 CQKLWTIEAI SLLDLISLKG KFSITALING DNQSIDISKP IRLMEGQTHA QADYLLALNS LKLLYKEYAG IGHKLKGTET YISRDMQFMS KTIQHNGVYY PASIKKVLRV GPWINTILDD FKVSLESIGS
 911 LTQELEYRGE SLLCSLIFRN VWLYNQIALQ LKNHALCNNK LYLDILKVLK HLKTFFNLDN IDTALTLYMN LPMLFGGGDP NLLYRSFYRR TPDFLTEAIV HSVFILSYYT NHDLKDKLQD LSDDRLNKFL
1041 TCIITFDKNP NAEFVTLMRD PQALGSERQA KITSEINRLA VTEVLSTAPN KIFSKSAQHY TTTEIDLNDI MQNIEPTYPH GLRVVYESLP FYKAEKIVNL ISGTKSITNI LEKTSAIDLT DIDRATEMMR
1171 KNITLLIRIL PLDCNRDKRE ILSMENLSIT ELSKYVRERS WSLSNIVGVT SPSIMYTMDI KYTTSTISSG IIIEKYNVNS LTRGERGPTK PWVGSSTQEK KTMPVYNRQV LTKKQRDQID LLAKLDWVYA
1301 SIDNKDEFME ELSIGTLGLT YEKAKKLFPQ YLSVNYLHRL TVSSRPCEFP ASIPAYRTTN YHFDTSPINR ILTEKYGDED IDIVFQNCIS FGLSLMSVVE QFTNVCPNRI ILIPKLNEIH LMKPPIFTGD
1431 VDIHKLKQVI QKQHMFLPDK ISLTQYVELF LSNKTLKSGS HVNSNLILAH KISDYFHNTY ILSTNLAGHW ILIIQLMKDS KGIFEKDWGE GYITDHMFIN LKVFFNAYKT YLLCFHKGYG KAKLECDMNT
1561 SDLLCVLELI DSSYWKSMSK VFLEQKVIKY ILSQDASLHR VKGCHSFKLW FLKRLNVAEF TVCPWVVNID YHPTHMKAIL TYIDLVRMGL INIDRIHIKN KHKFNDEFYT SNLFYINYNF SDNTHLLTKH
1691 IRIANSELEN NYNKLYHPTP ETLENILANP IKSNDKKTLN DYCIGKNVDS IMLPLLSNKK LIKSSAMIRT NYSKQDLYNL FPMVVIDRII DHSGNTAKSN QLYTTTSHQI SLVHNSTSLY CMLPWHHINR
1821 FNFVFSSTGC KISIEYILKD LKIKDPNCIA FIGEGAGNLL LRTVVELHPD IRYIYRSLKD CNDHSLPIEF LRLYNGHINI DYGENLTIPA TDATNNIHWS YLHIKFAEPI SLFVCDAELS VTVNWSKIII
1951 EWSKHVRKCK YCSSVNKCML IVKYHAQDDI DFKLDNITIL KTYVCLGSKL KGSEVYLVLT IGPANIFPVF NVVQNAKLIL SRTKNFIMPK KADKESIDAN IKSLIPFLCY PITKKGINTA LSKLKSVVSG
2081 DILSYSIAGR NEVFSNKLIN HKHMNILKWF NHVLNFRSTE LNYNHLYMVE STYPYLSELL NSLTTNELKK LIKITGSLLY NFHNE
```

b Mass spec coverage of the RSV P protein

```
     1          11         21         31         41         51         61         71         81         91         101        111        121
   1 MEKFAPEFHG EDANNRATKF LESIKGKFAS PKDPKKKDSI ISVNSIDIEV TKESPITSNS TIINPTNETD DTAGNKPNYQ RKPLVSFKED PTPSDNPFSK LYKETIETFD NNEEESSYSY EEINDQTNDN
 131 ITARLDRIDE KLSEILGMLH TLVVASAGPT SARDGIRDAM VGLREEMIEK IRTEALMTND RLEAMARLRN EESEKMAKDT SDEVSLNPTS EKLNNLLEGN DSDNDLSLED F
```

Supplementary Figure 5: The mass spectrometry analysis of the RSV polymerase (L:P) complex. **a**, The identified polypeptides cover 90% of the RSV L protein as highlighted in gray. **b**, The identified polypeptides cover 93% of the RSV P protein as highlighted in gray.

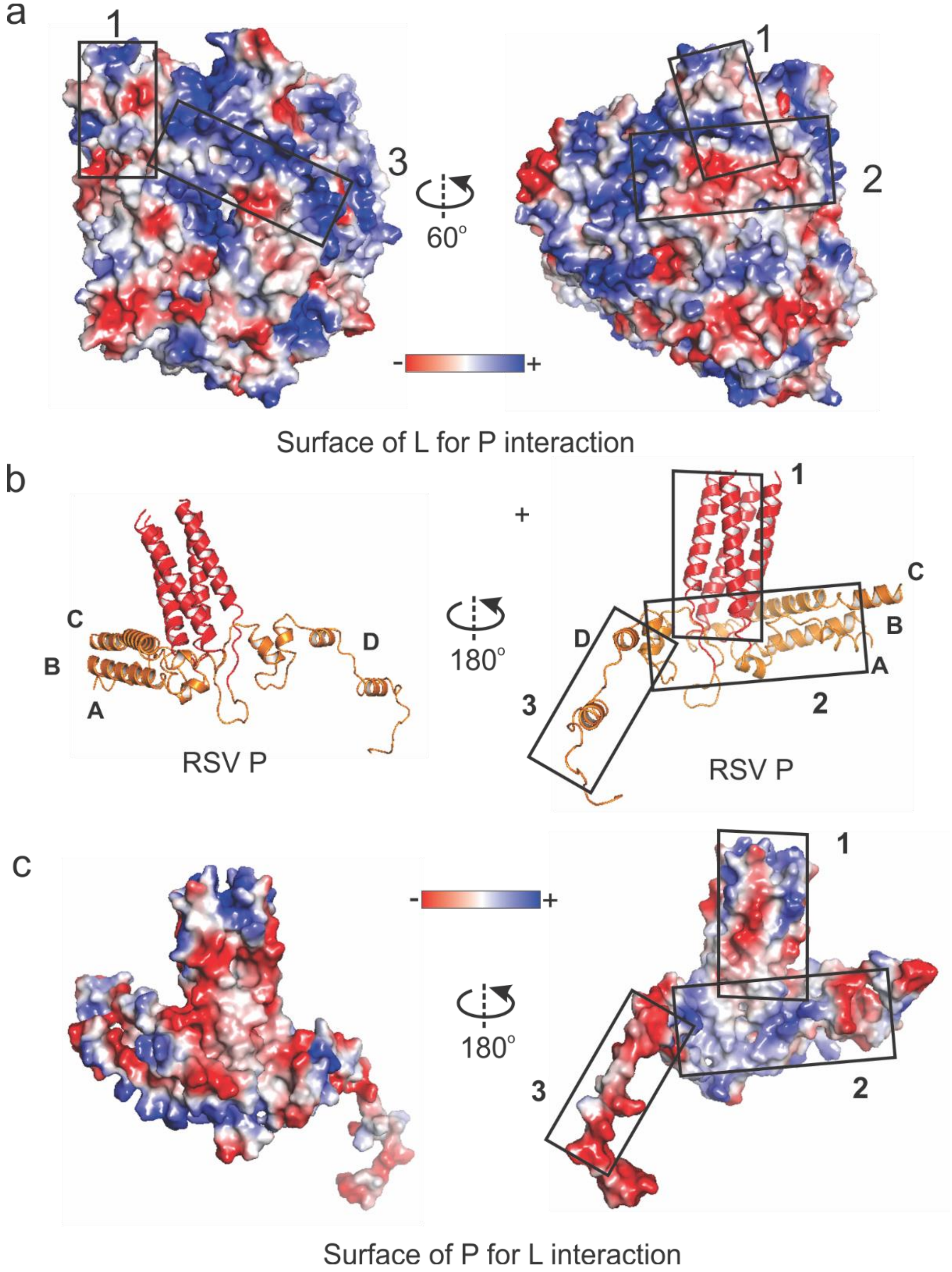

Supplementary Figure 6: Electrostatic surface for L and P interactions. a, Electrostatic surface potential of the surface of L for P interaction is divided into three regions as shown in boxes 1, 2, and 3 (see text for details). **b**, the cartoon representation of the RSV P in two views. The oligomerization domain (P_{OD}, red) shows a four-helix bundle, and the C-terminal domain (P_{CTD}, orange) arrange different for each of the four chains, A, B, C, and D. **c**, Electrostatic surface potential of the surface of P for L interaction. The corresponding L interaction regions of P are shown in boxes 1, 2, and 3.

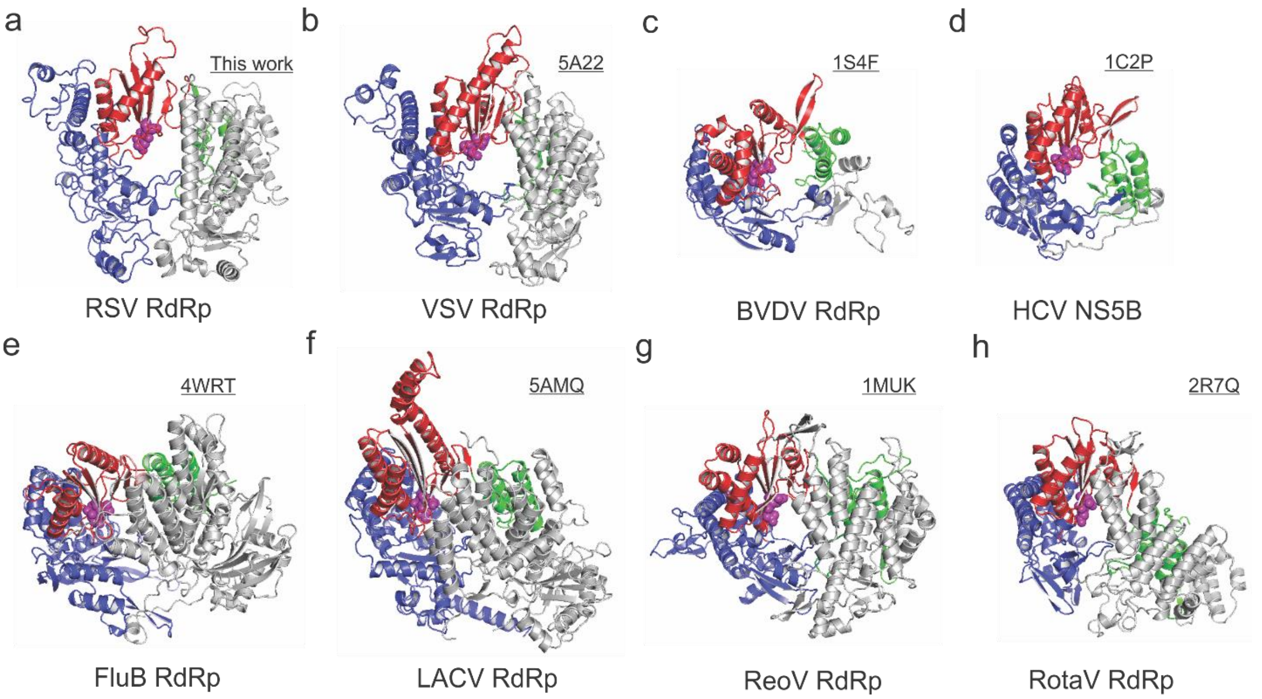

Supplementary Figure 7: Structural superimposition of the RNA dependent RNA polymerase (RdRp) domain. The conventional “fingers-palm-thumb” right-hand motifs are displayed as the fingers, blue; the palm, red; and the thumb, green. The rest of the domain is gray. The superimposed structures are selected based on the DALI structural similarity search. **a**, The RdRp of the RSV L (this work). **b**, The RdRp of vesicular stomatitis virus (VSV) (PDB: 5A22 [http://dx.doi.org/10.2210/pdb5A22/pdb]). **c**, The RdRp of bovine viral diarrhea virus (BVDV) (PDB: 1S4F [http://dx.doi.org/10.2210/pdb1S4F/pdb]). **d**, The RdRp of hepatitis C virus NS5B (PDB: 1C2P [http://dx.doi.org/10.2210/pdb1C2P/pdb]). **e**, The RdRp of Influenza B (PDB: 4WRT [http://dx.doi.org/10.2210/pdb4WRT/pdb]). **f**, The RdRp of La Crosse Bunyavirus (PDB: 5AMQ [http://dx.doi.org/10.2210/pdb5AMQ/pdb]). **g**, The RdRp of reovirus lambda3 (PDB: 1MUK [http://dx.doi.org/10.2210/pdb1MUK/pdb]). **h**, The RdRp of rotavirus (PDB: 2R7Q [http://dx.doi.org/10.2210/pdb2R7Q/pdb]). The PDB accession codes are underlined.

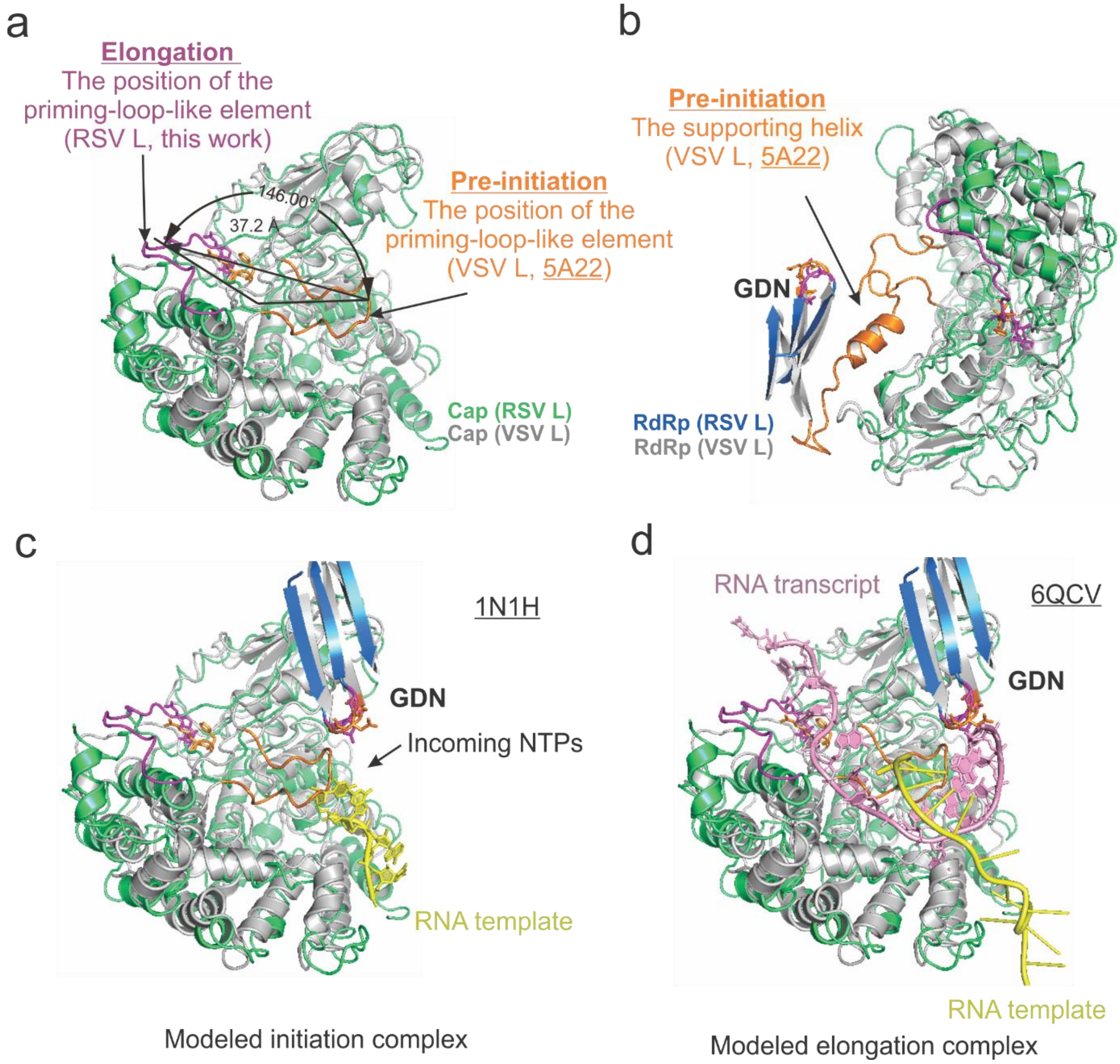

Supplementary Figure 8: Structural comparison of the capping domain (Cap). a, The distance (37.2 Å) and conformational rearrangement (146°) of the priming-loop-like element from RSV L (purple) and VSV L (orange). **b**, The relative position of the RdRp active site (GDN, RSV, magenta; VSV, orange) and supporting helix (VSV, orange) with respect to the priming-loop-like element of RSV L (blue) and VSV L (gray). **c**, The Cap domain with overlaid RNA from Reo initiation complex (PDB: 1N1H [http://dx.doi.org/10.2210/pdb1N1H/pdb]). The RNA template is colored in yellow, and the incoming NTPs are indicated with an arrow. **d**, The Cap domain with overlaid RNA from FluB elongation complex (PDB: 6QCV [http://dx.doi.org/10.2210/pdb6QCV/pdb]). The RNA template is in yellow, and the RNA transcript is in pink. As expected, the position of the priming-loop-like element of the RSV L accommodates the RNA transcript well, while the position of the priming-loop-like element of the VSV L collides with the RNA transcript, suggesting the priming-loop-like element is out of the way after initiation and during elongation. The PDB accession codes are underlined.

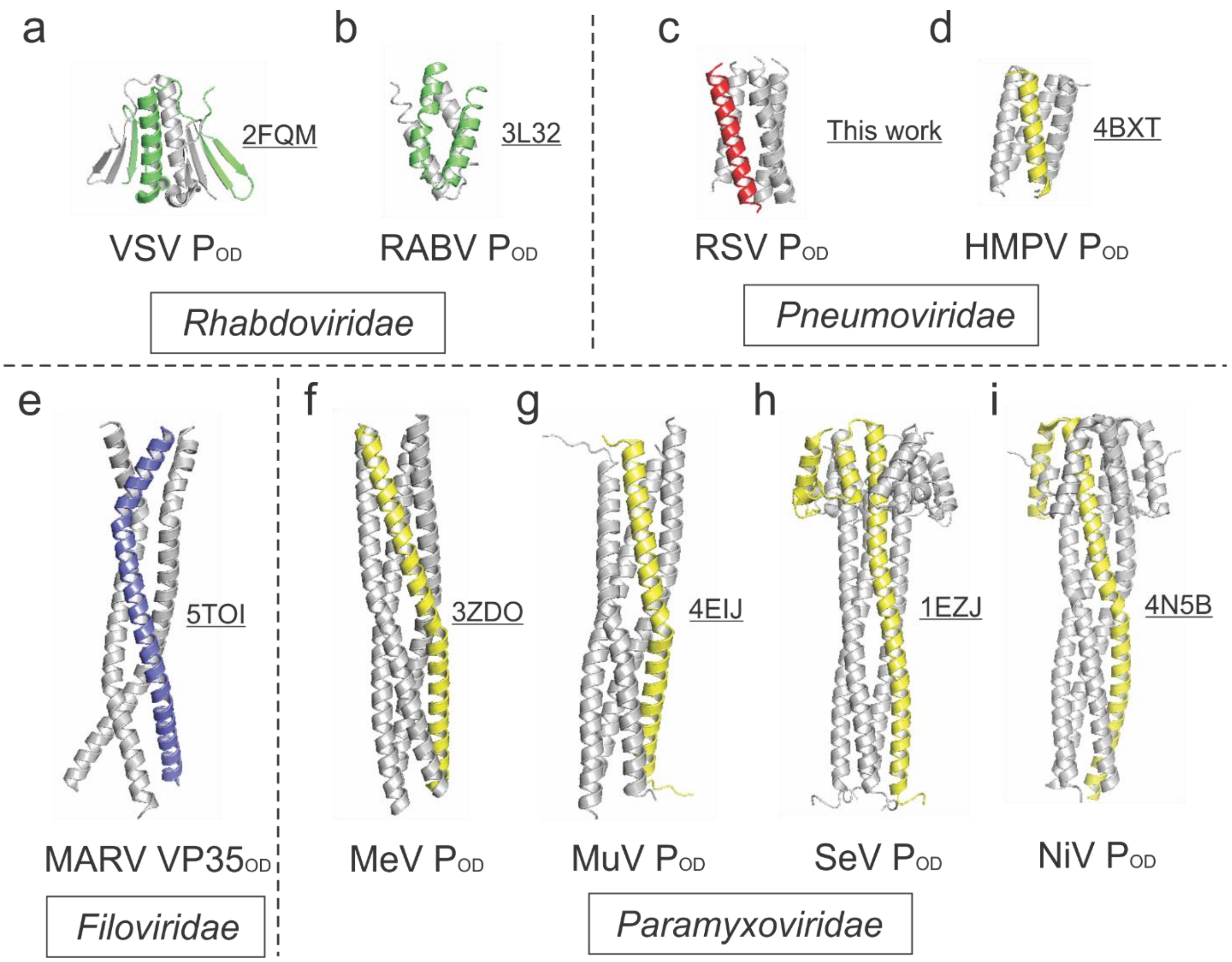

Supplementary Figure 9: Structural comparison of the P_{OD} domain from the order of *Mononegavirales*. One monomer of the dimmer, trimer and tetramer of the P_{OD} is colored in light green, light blue, and light yellow, respectively. One monomer of the RSV P_{OD} (this work) is colored in red. **a-b**, Dimmer form. *Rhabdoviridae*: vesicular stomatitis virus (VSV) (PDB: 2FQM [http://dx.doi.org/10.2210/pdb2FQM/pdb]) and rabies virus (RABV) (PDB: 3L32 [http://dx.doi.org/10.2210/pdb3L32/pdb]). **c-d**, Short tetramer form. *Pneumoviridae*: respiratory syncytial virus (RSV) (This work) and human metapneumovirus (hMPV) (PDB: 4BXT [http://dx.doi.org/10.2210/pdb4BXT/pdb]). **e**, Trimer form. *Filoviridae*: Marburg virus (MARV) (PDB: 5TOI [http://dx.doi.org/10.2210/pdb5TOI/pdb]). **f-i**, Long tetramer form. *Paramyxoviridae*: Measles virus (MeV) (PDB: 3ZDO [http://dx.doi.org/10.2210/pdb3ZDO/pdb]), Mumps virus (MuV) (PDB: 4EIJ [http://dx.doi.org/10.2210/pdb4EIJ/pdb]), Sendai virus (SeV) (PDB: 1EZJ [http://dx.doi.org/10.2210/pdb1EZJ/pdb]), and Nipah virus (NiV) (PDB: 4N5B [http://dx.doi.org/10.2210/pdb4N5B/pdb]). The PDB accession codes are underlined.

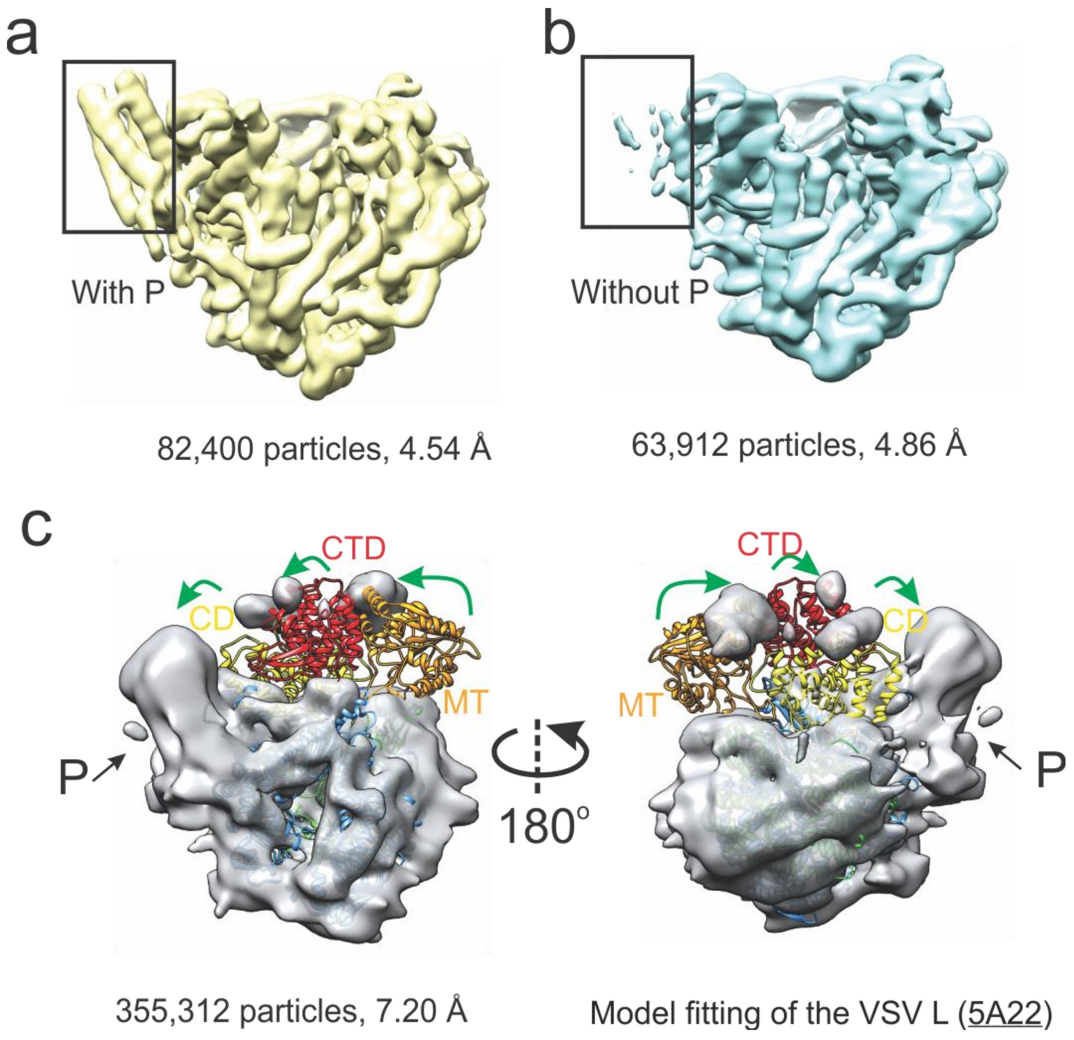

Supplementary Figure 10: Conformational flexibility of the RSV polymerase (L:P) and the potential arrangement of the missing domains. a-b, Different occupy of the P protein with respect to the L protein: L with P (**a**) and without P (**b**). The particles and the resolution of the map are shown below. **c**, The potential arrangement of the missing domains of the RSV L. The 7.2 Å map shows additional density on the top of the map. The superimposition of the VSV L into the map reveals the possible rearrangement of the missing domains (green arrow). The domain of the VSV L is colored as follows: RdRp (blue), Cap (green), CD (yellow), MT (orange), and CTD (red). The location of P is shown with the black arrow.

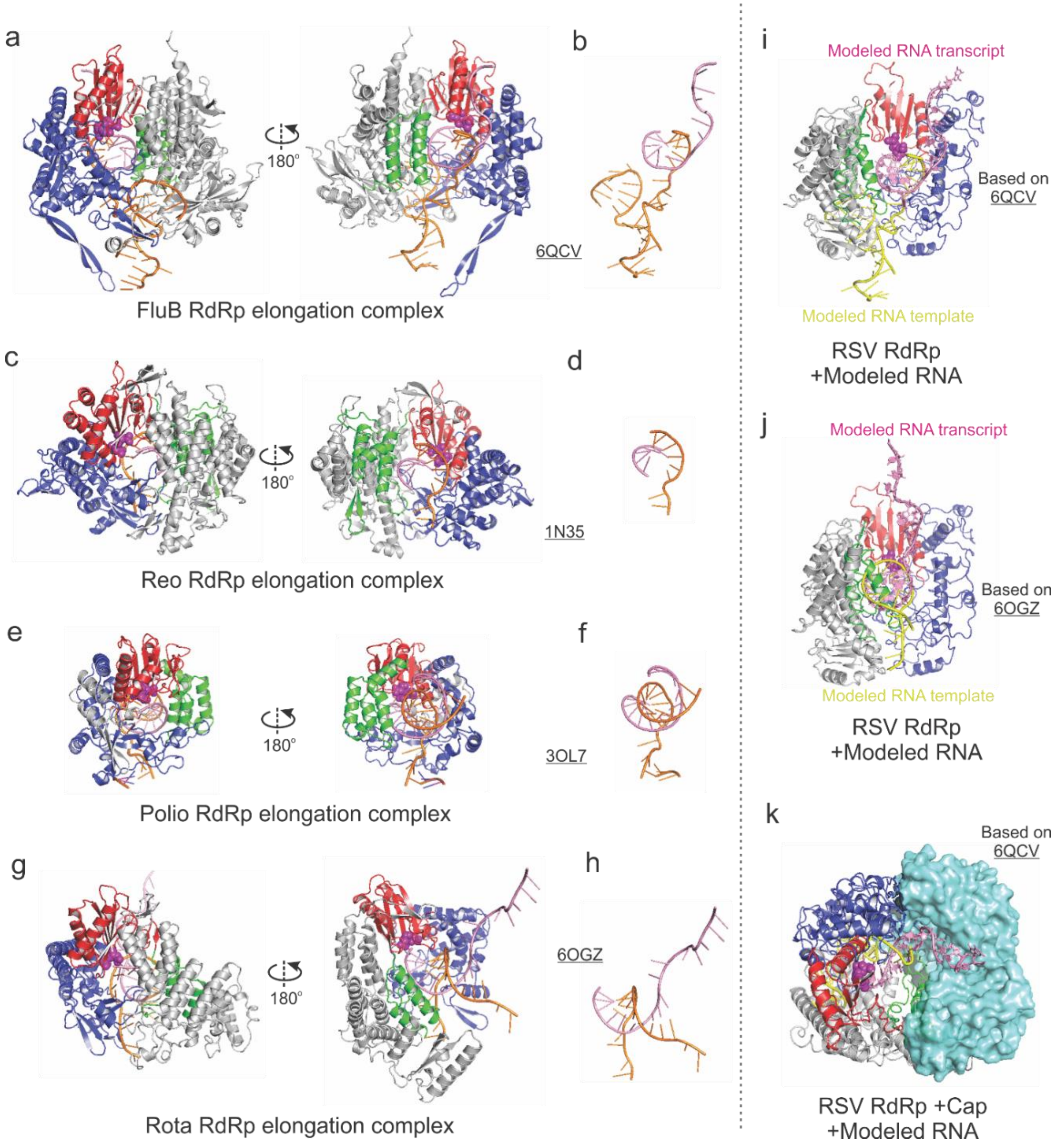

Supplementary Figure 11: Structural comparison and the modeling of the elongation complex. The conventional “fingers-palm-thumb” right-hand motifs are displayed as the fingers (blue), the palm (red), and the thumb (green). The rest of the proteins are in gray. The RNA template (orange) and the RNA transcript (pink) are shown. **a-b**, The influenza B elongation complex (**a**) with RNA template and transcript (**b**) (PDB: 6QCV [http://dx.doi.org/10.2210/pdb6QCV/pdb]). **c-d**, The Reovirus elongation complex (**c**) with RNA template and transcript (**d**) (PDB: 1N35 [http://dx.doi.org/10.2210/pdb1N35/pdb]). **e-f**, The Polio elongation complex (**e**) with RNA template and transcript (**f**) (PDB: 3OL7 [http://dx.doi.org/10.2210/pdb3OL7/pdb]). **g-h**, The Rota elongation complex (**g**) with RNA template and transcript (**h**) (PDB: 6OGZ [http://dx.doi.org/10.2210/pdb6OGZ/pdb]). **i**,**j**, The modeled RNA template (yellow) and transcript (pink) with the RdRp of RSV. **k**, The RSV RdRp and Cap (cyan surface) with the modeled RNAs. The space between the RdRp and Cap domains is sufficient to allow RNA transcript passing through. The PDB accession codes are underlined.

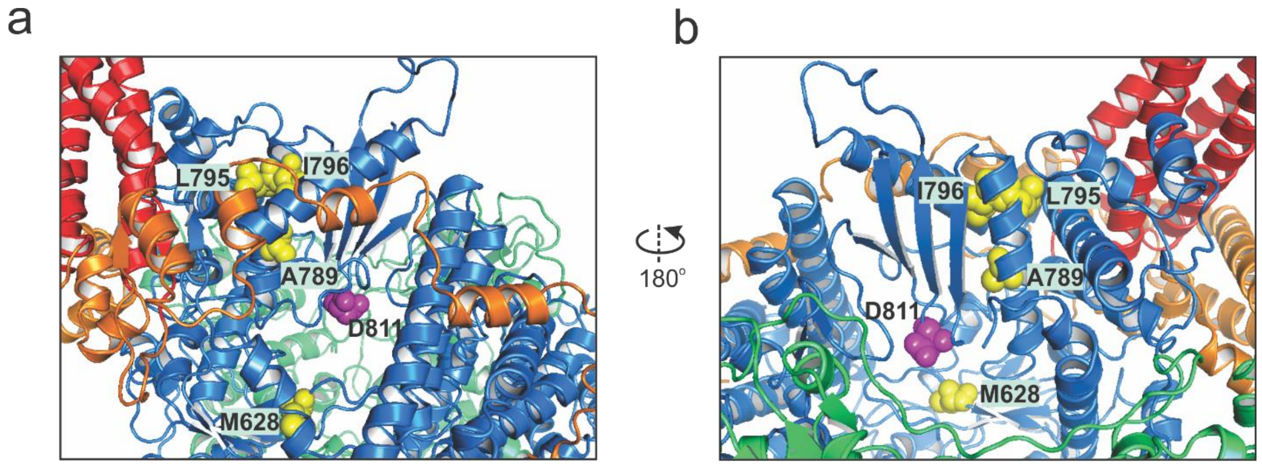

Supplementary Figure 12: The implications of the RSV polymerase inhibitors. a-b, The locations of the QUAD mutations (M628L, A789V, L795I, and I796V) of nucleoside analog inhibitor ALS-8112 and the active site residue (D811). The QUAD mutations are in yellow spheres, and the active site residue D811 is magenta spheres.

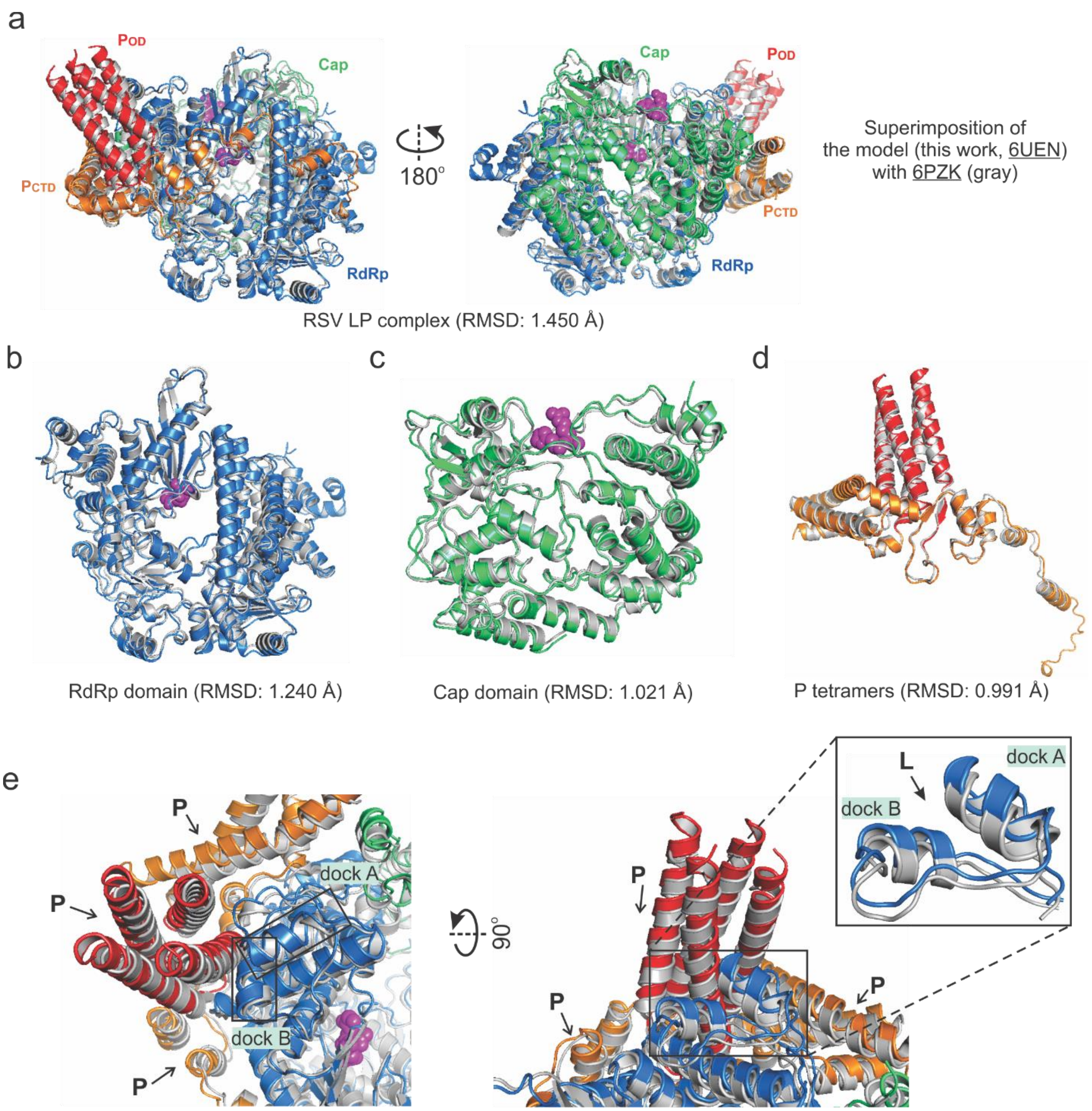

Supplementary Figure 13: The structure comparison of the model of this work (PDB: 6UEN) with recent published RSV L:P complex (PDB: 6PZK). The model of this work is in the same color as Fig. 1a, and Fig. 2. The published RSV L:P complex is colored in gray. **a,** The superimposition of the entire model of this work (6UEN [http://dx.doi.org/10.2210/pdb6UEN/pdb]) with RSV L:P complex (6PZK [http://dx.doi.org/10.2210/pdb6PZK/pdb]) with an RMSD value of 1.450 Å. **b-d**, The superimposition of the individual RdRp domain, Cap domain, and P tetramers with the RMSD values of 1.240 Å, 1.021 Å, and 0.991 Å, respectively. The RMSD values are indicated. **e**, The difference between the interface of the L:P complex. The superimposition is based on the RdRp domain. The arrows indicate the shift direction of P or L of (6PZK [http://dx.doi.org/10.2210/pdb6PZK/pdb]) with respect to the model of this work (6UEN [http://dx.doi.org/10.2210/pdb6UEN/pdb]). Dock A and dock B, the two helixes of L (6PZK [http://dx.doi.org/10.2210/pdb6PZK/pdb]) that interact with P, adopt more compact packing than that of the model of this work (6UEN [http://dx.doi.org/10.2210/pdb6UEN/pdb]). The PDB accession codes are underlined.

**Data collection and Processing**	
Magnification	130,000
Voltage (kV)	200
Electron Exposure (e-/Å2)	54.6
Defocus range (µm)	-1.25 ~ -2.75
Pixel size (Å)	1.045
Symmetry imposed	C1
Initial particle images (no.)	792,070
Final particle images (no.)	253,372
Map resolution (Å)	3.67
FSC threshold	0.143
**Refinement**	
B factor (Å) (min/max/mean)	20.49/128.54/58.94
Model composition	
Chains	5
Protein residues	1696
Non-hydrogen atoms	13705
R.M.S. deviations	
Bond lengths (Å) (# > 4σ)	0.006 (0)
Bond angles (°) (# > 4σ)	0.642 (0)
Validation	
MolProbity score	1.80
Clashscore	8.93
CaBLAM outliers (%)	2.52
CC (model to map)	0.83
Ramachandran statistics	
Favored	95.36
Allowed	4.64
Outliers	0

Supplementary Table 1: Cryo-EM data collection, refinement, and validation statistics.

a. The DNA sequence of the codon-optimized RSV L gene:

ATGGACCCCATCATCAACGGCAACAGCGCCAACGTGTACCTGACCGACAGCTACCTGAAGGGC
GTGATCAGCTTCAGCGAGTGCAACGCCCTGGGCAGCTACATCTTCAACGGCCCCTACCTGAAG
AACGACTACACCAACCTGATCAGCCGGCAGAACCCCCTGATCGAGCACATGAACCTGAAGAAG
CTGAACATCACCCAGAGCCTGATCAGCAAGTACCACAAGGGCGAGATCAAGCTGGAAGAGCCC
ACCTACTTCCAGAGCCTGCTGATGACCTACAAGAGCATGACCAGCAGCGAGCAGATCGCCACC
ACCAACCTGCTGAAGAAGATCATCAGGCGGGCCATCGAGATCAGCGACGTGAAGGTGTACGCC
ATCCTGAACAAGCTGGGCCTGAAAGAGAAGGACAAGATCAAGAGCAACAACGGCCAGGACGAG
GACAACAGCGTGATCACCACCATCATCAAGGACGACATCCTGAGCGCCGTGAAGGACAACCAG
AGCCACCTGAAGGCCGACAAGAACCACAGCACCAAGCAGAAGGACACCATCAAGACCACCCTG
CTGAAAAAGCTGATGTGCAGCATGCAGCACCCCCCCAGCTGGCTGATCCACTGGTTCAACCTGT
ACACCAAGCTGAACAACATCCTGACCCAGTACCGGTCCAACGAGGTGAAGAACCACGGCTTCA
CCCTGATCGACAACCAGACCCTGAGCGGCTTCCAGTTCATCCTGAATCAGTACGGCTGCATCGT
GTACCACAAAGAGCTGAAGCGGATCACCGTGACCACCTACAACCAGTTTCTGACCTGGAAGGA
CATCAGCCTGAGCCGGCTGAACGTGTGCCTGATCACCTGGATCAGCAACTGCCTGAACACCCT
CAACAAGTCTCTGGGCCTGCGGTGCGGCTTCAACAACGTGATCCTGACACAGCTGTTTCTGTAC
GGCGACTGCATCCTGAAGCTGTTCCACAACGAGGGCTTCTACATCATCAAAGAGGTGGAGGGC
TTCATCATGAGCCTGATCCTGAATATCACCGAAGAGGACCAGTTCCGGAAGCGGTTCTACAACA
GCATGCTGAATAACATCACCGACGCCGCCAACAAGGCCCAGAAGAACCTGCTGTCCAGAGTGT
GCCACACCCTGCTGGACAAGACCGTGAGCGACAACATCATCAATGGCCGGTGGATCATCCTGC
TGTCTAAGTTTCTGAAGCTGATTAAACTGGCCGGCGACAACAACCTGAACAATCTGAGCGAGCT
GTACTTCCTGTTCCGGATCTTCGGCCACCCCATGGTGGACGAGCGGCAGGCCATGGACGCCGT
GAAGATCAACTGCAACGAGACCAAGTTCTATCTGCTGTCCTCCCTGAGCATGCTGAGGGGCGC
CTTCATCTACCGGATCATCAAGGGCTTCGTGAACAACTACAACCGGTGGCCCACCCTGCGGAAC
GCCATCGTGCTGCCCCTGCGGTGGCTGACCTACTACAAGCTGAATACCTACCCCAGCCTGCTG
GAACTGACAGAGCGGGACCTGATCGTGCTGTCCGGCCTGCGGTTCTACCGGGAGTTCCGGCT
GCCCAAGAAGGTGGACCTGGAAATGATCATCAACGACAAGGCCATCAGCCCCCCCAAGAACCT
GATCTGGACCAGCTTCCCCCGGAACTACATGCCCAGCCACATCCAGAACTACATCGAGCACGA
GAAGCTGAAGTTCAGCGAGAGCGACAAGAGCAGGCGGGTGCTGGAATACTACCTGCGGGACA
ACAAGTTCAACGAGTGCGACCTGTACAACTGCGTGGTGAACCAGTCCTACCTGAACAACCCCAA
CCACGTGGTGTCCCTGACCGGCAAAGAGCGGGAGCTGTCCGTCGGCCGGATGTTCGCCATGC
AGCCCGGCATGTTCCGGCAGGTGCAGATCCTGGCCGAGAAGATGATCGCCGAGAACATCCTGC
AGTTCTTCCCCGAGAGCCTGACCAGATACGGCGACCTGGAACTGCAGAAGATCCTGGAACTGA
AGGCCGGCATCAGCAACAAGAGCAACCGGTACAACGACAACTACAATAACTACATCAGCAAGTG
CAGCATCATCACCGACCTGAGCAAGTTCAACCAGGCCTTCAGATACGAGACCAGCTGCATCTGC
AGCGACGTGCTGGACGAGCTGCACGGCGTGCAGAGCCTGTTCTCCTGGCTGCACCTGACCATC
CCCCACGTGACCATCATCTGCACCTACCGGCACGCCCCTCCCTACATCGGCGACCACATCGTG
GACCTGAATAACGTGGACGAGCAGAGCGGCCTGTACAGATACCACATGGGCGGCATCGAGGG
CTGGTGCCAGAAGCTGTGGACCATCGAGGCCATCTCCCTGCTGGATCTGATCAGCCTGAAGGG
CAAGTTCTCCATCACCGCCCTGATCAACGGCGACAACCAGTCCATCGACATCTCCAAGCCCATC
CGGCTGATGGAAGGCCAGACCCACGCCCAGGCCGACTACCTGCTGGCCCTGAATAGCCTGAA
GCTGCTGTACAAAGAGTACGCCGGCATCGGCCACAAGCTGAAGGGCACCGAGACCTACATCAG
CCGGGACATGCAGTTCATGAGCAAGACCATCCAGCACAACGGCGTGTACTACCCCGCCAGCAT
CAAGAAAGTGCTGAGAGTGGGCCCCTGGATCAACACCATCCTGGACGACTTCAAGGTGTCCCT
GGAAAGCATCGGCAGCCTGACCCAGGAACTGGAATACCGGGGCGAGTCTCTGCTGTGCAGCCT
GATCTTCCGGAACGTGTGGCTGTACAACCAGATCGCCCTGCAGCTGAAGAATCACGCCCTGTG
CAACAACAAGCTGTACCTGGACATCCTGAAGGTGCTGAAGCACCTGAAAACCTTTTTCAACCTG
GACAACATCGATACCGCCCTGACCCTGTACATGAATCTGCCCATGCTGTTTGGCGGCGGAGAC
CCCAATCTGCTGTACCGGTCCTTCTACCGGCGGACCCCCGACTTCCTGACCGAGGCCATCGTG
CACAGCGTGTTCATCCTGTCCTACTACACCAATCACGACTTGAAGGACAAGCTGCAGGACCTGA
GCGACGACCGGCTGAATAAGTTCCTGACCTGTATCATCACCTTCGACAAGAATCCCAACGCCGA

GTTCGTGACCCTGATGCGGGACCCCCAGGCCCTGGGCTCCGAGAGGCAGGCCAAGATCACCA
GCGAGATCAACCGGCTGGCCGTGACCGAGGTGCTGTCCACCGCCCCCAACAAGATCTTCAGCA
AGAGCGCCCAGCACTACACCACCACCGAGATCGACCTGAACGACATCATGCAGAACATCGAGC
CTACCTACCCCCACGGCCTGCGGGTGGTGTACGAGAGCCTGCCCTTCTACAAGGCCGAGAAAA
TCGTGAATCTGATCTCCGGCACCAAGAGCATCACCAACATCCTGGAAAAGACCAGCGCCATCGA
CCTGACCGATATCGACCGGGCCACCGAGATGATGCGGAAGAATATCACACTGCTGATCAGAAT
CCTGCCCCTGGACTGCAACCGGGACAAGCGGGAGATCCTGAGCATGGAAAACCTGAGCATCAC
CGAGCTGTCCAAGTACGTGCGGGAGCGGAGCTGGTCCCTGAGCAACATCGTGGGCGTGACCA
GCCCCAGCATCATGTACACCATGGACATCAAGTACACCACCTCCACCATCAGCAGCGGCATCAT
CATCGAGAAGTACAACGTGAACTCCCTGACCAGGGGCGAGAGGGGCCCCACCAAGCCCTGGG
TGGGCAGCAGCACCCAGGAAAAGAAAACCATGCCCGTGTACAACAGGCAGGTGCTGACCAAGA
AGCAGAGGGACCAGATCGATCTGCTGGCCAAGCTGGACTGGGTGTACGCCTCCATCGACAACA
AGGACGAGTTCATGGAAGAGCTGTCCATCGGCACCCTGGGCCTGACCTACGAGAAGGCCAAGA
AGCTGTTTCCCCAGTACCTGAGCGTGAACTACCTGCACAGGCTGACCGTGAGCAGCCGGCCCT
GCGAGTTCCCCGCCTCCATCCCCGCCTACCGGACCACCAACTACCACTTCGACACCAGCCCCA
TCAACCGGATTCTGACCGAGAAGTACGGCGACGAGGACATCGACATCGTGTTCCAGAACTGCA
TCAGCTTCGGCCTGAGCCTGATGAGCGTGGTGGAGCAGTTCACCAACGTGTGCCCCAACAGAA
TCATCCTGATCCCCAAGCTGAATGAGATCCACCTGATGAAGCCCCCCATCTTCACCGGCGACGT
GGATATCCACAAACTGAAGCAGGTGATCCAGAAACAGCACATGTTTCTGCCCGACAAGATCTCC
CTGACACAGTACGTGGAGCTGTTCCTGTCCAACAAGACCCTGAAGAGCGGCAGCCACGTGAAC
AGCAACCTGATCCTGGCCCACAAGATCAGCGATTACTTCCACAACACCTACATCCTGTCCACCA
ATCTGGCCGGCCACTGGATTCTGATCATCCAGCTGATGAAAGACAGCAAGGGCATCTTCGAGAA
GGACTGGGGCGAGGGCTACATCACCGATCACATGTTCATCAACCTGAAGGTGTTCTTCAACGCC
TACAAGACCTACCTGCTGTGCTTCCACAAGGGCTACGGCAAGGCCAAACTGGAATGCGACATG
AACACCAGCGATCTGCTGTGCGTGCTGGAACTGATCGACAGCAGCTACTGGAAGAGCATGAGC
AAAGTGTTTCTGGAACAGAAGGTGATCAAGTATATCCTGAGCCAGGACGCCAGCCTGCACCGG
GTGAAGGGCTGCCACTCTTTCAAGCTCTGGTTCCTGAAGAGACTGAACGTGGCCGAGTTCACC
GTGTGCCCTTGGGTGGTGAACATCGACTACCACCCCACCCACATGAAGGCCATCCTGACCTAC
ATCGACCTGGTCCGGATGGGCCTGATCAACATCGACCGCATCCACATCAAGAACAAGCACAAGT
TCAATGACGAGTTCTACACCAGCAACCTGTTCTACATCAACTACAACTTCAGCGACAACACCCAC
CTGCTGACAAAGCACATCCGGATCGCCAACAGCGAGCTGGAAAACAACTATAATAAGCTGTACC
ACCCTACCCCCGAGACCCTGGAAAACATCCTGGCCAACCCCATCAAGTCCAACGACAAGAAAAC
CCTGAACGACTACTGCATCGGCAAGAACGTGGACAGCATCATGCTGCCTCTGCTGTCCAATAAG
AAGCTGATTAAGAGCAGCGCCATGATCCGGACCAACTACAGCAAGCAGGATCTGTACAACCTGT
TCCCTATGGTGGTGATCGACAGGATCATCGACCACAGCGGCAATACCGCCAAGTCCAACCAGC
TGTACACCACAACCAGCCACCAGATCAGCCTGGTGCACAACAGCACCAGCCTGTACTGCATGCT
GCCCTGGCACCACATCAACCGGTTCAACTTCGTGTTCAGCAGCACCGGCTGCAAGATCAGCAT
CGAGTACATCCTGAAAGACCTGAAGATCAAGGACCCCAACTGCATCGCCTTCATCGGCGAAGG
CGCCGGAAACCTGCTGCTGCGGACAGTGGTGGAGCTGCACCCCGACATCCGGTACATCTACAG
AAGCCTGAAGGACTGCAACGACCACAGCCTGCCTATCGAGTTCCTGAGACTGTACAACGGCCA
CATCAATATCGACTACGGCGAGAACCTGACAATCCCCGCCACCGATGCCACCAACAACATCCAC
TGGTCTTACCTGCACATCAAGTTCGCCGAGCCCATCAGCCTGTTCGTGTGCGACGCCGAGCTG
TCTGTGACCGTGAACTGGTCCAAGATCATCATTGAGTGGAGCAAGCACGTGCGGAAGTGCAAG
TACTGCAGCAGCGTGAACAAGTGCATGCTGATCGTGAAGTACCACGCCCAGGACGATATCGAC
TTCAAGCTGGACAATATCACCATCCTGAAAACATATGTGTGCCTGGGCAGCAAGCTGAAAGGCA
GCGAAGTGTACCTGGTGCTGACAATCGGCCCTGCCAACATCTTCCCCGTGTTCAACGTGGTGC
AGAACGCCAAGCTGATCCTGAGCCGCACCAAGAACTTCATCATGCCTAAGAAGGCCGATAAAGA
GAGCATCGACGCCAACATCAAGTCCCTGATCCCTTTCCTGTGCTACCCCATCACCAAGAAGGGC
ATCAACACCGCCCTGTCCAAGCTGAAGTCCGTGGTGTCCGGCGACATCCTGTCTTACAGCATCG
CCGGCAGAAACGAGGTGTTCTCCAACAAACTGATCAACCACAAGCACATGAATATCCTGAAGTG
GTTCAACCACGTGCTGAACTTCAGAAGCACCGAGCTGAACTACAACCACCTGTACATGGTGGAG

AGCACCTACCCCTACCTGTCCGAGCTGCTGAACAGCCTGACCACCAATGAGCTGAAGAAACTGA TCAAGATCACCGGCAGCCTGCTGTATAACTTCCACAATGAGTAA

b. The DNA sequence of the codon-optimized RSV P gene:

ATGGAGAAGTTCGCCCCCGAGTTCCACGGCGAGGACGCCAACAACCGGGCCACCAAGTTTCTG GAGAGCATCAAGGGCAAGTTCACCAGCCCCAAGGACCCCAAGAAGAAGGACAGCATCATCAGC GTGAACAGCATCGACATCGAGGTGACCAAGGAGAGCCCCATCACCAGCAACAGCACCATCATC AACCCCACCAACGAGACCGACGACACCGCCGGCAACAAGCCCAACTACCAGCGGAAGCCCCT GGTGTCCTTCAAGGAGGACCCCACCCCCAGCGACAACCCCTTCAGCAAGCTGTACAAGGAGAC CATCGAGACCTTCGACAACAACGAGGAGGAGAGCAGCTACAGCTACGAGGAGATCAACGACCA GACCAACGACAACATCACCGCCAGGCTGGACCGGATCGACGAGAAGCTGTCCGAGATCCTGG GCATGCTGCACACCCTGGTGGTGGCCAGCGCCGGACCCACCTCCGCCAGGGACGGCATCCGG GACGCCATGGTGGGCCTGAGGGAGGAGATGATCGAGAAGATCCGGACCGAGGCCCTGATGAC CAACGACCGGCTGGAGGCCATGGCCCGGCTGAGGAACGAGGAGAGCGAGAAGATGGCCAAG GACACCAGCGACGAGGTGTCCCTGAACCCCACCTCCGAGAAGCTGAACAACCTGCTGGAGGG CAACGACAGCGACAACGACCTGAGCCTGGAGGACTTCTAA

c. The list of primer sequences:

**v1-RSVL_f_1**	5'-TACTTCCAATCCAATGCAATGGACCCCATCATCAACGG-3'
**v1-RSVL_rv_2165**	5'-TTATCCACTTCCAATGTTATTACTCATTGTGGAAGTTATACAGCAGG-3'
**v2-RSVP_f_1**	5'-TTTAAGAAGGAGATATAGATCATGGAGAAGTTCGCCCCC-3'
**v2-RSVP_rv_241**	5'-TTATGGAGTTGGGATCTTATTAGAAGTCCTCCAGGCTCAGGTC-3'
**RSVL-D811A-S**	5'-GATCAACGGCGCCAACCAGTCCATCGACATCTCC-3'
**RSVL-D811A-AN**	5'-GGACTGGTTGGCGCCGTTGATCAGGGCGGTGATGG-3'

Supplementary Table 2: a, The DNA sequence of the codon-optimized RSV L gene. **b,** The DNA sequence of the codon-optimized RSV P gene. **c,** The list of primer sequences used for cloning.
